# The E262K mutation in Lamin A links nuclear proteostasis imbalance to laminopathy‐associated premature aging

**DOI:** 10.1111/acel.13688

**Published:** 2022-10-12

**Authors:** Debasish Kumar Ghosh, Shruti Pande, Jeevan Kumar, Dhanya Yesodharan, Sheela Nampoothiri, Periyasamy Radhakrishnan, Chilakala Gangi Reddy, Akash Ranjan, Katta M. Girisha

**Affiliations:** ^1^ Department of Medical Genetics Manipal Academy of Higher Education, Kasturba Medical College, Manipal Manipal India; ^2^ Department of Pediatric Genetics Amrita Institute of Medical Sciences & Research Centre Cochin India; ^3^ Suma Genomics Private Limited Manipal Center for Biotherapeutics Research and Department of Reproductive Science Manipal Academy of Higher Education Manipal India; ^4^ Computational and Functional Genomics Group Centre for DNA Fingerprinting and Diagnostics Hyderabad India; ^5^ Regional Centre for Biotechnology Faridabad India

**Keywords:** lamin A, laminopathy‐associated progeroid disorder, loss of DNA damage repair, nuclear proteostasis imbalance, protein aggregation, protein instability

## Abstract

Deleterious, mostly de novo, mutations in the lamin A (*LMNA*) gene cause spatio‐functional nuclear abnormalities that result in several laminopathy‐associated progeroid conditions. In this study, exome sequencing in a sixteen‐year‐old male with manifestations of premature aging led to the identification of a mutation, c.784G>A, in *LMNA*, resulting in a missense protein variant, p.Glu262Lys (E262K), that aggregates in nucleoplasm. While bioinformatic analyses reveal the instability and pathogenicity of LMNA^E262K^, local unfolding of the mutation‐harboring helical region drives the structural collapse of LMNA^E262K^ into aggregates. The E262K mutation also disrupts SUMOylation of lysine residues by preventing UBE2I binding to LMNA^E262K^, thereby reducing LMNA^E262K^ degradation, aggregated LMNA^E262K^ sequesters nuclear chaperones, proteasomal proteins, and DNA repair proteins. Consequently, aggregates of LMNA^E262K^ disrupt nuclear proteostasis and DNA repair response. Thus, we report a structure–function association of mutant LMNA^E262K^ with toxicity, which is consistent with the concept that loss of nuclear proteostasis causes early aging in laminopathies.

## Graphical abstract

Model of mutant LMNA‐mediated induction of nuclear proteotoxicity and DNA damage and its impact on the onset of laminopathy‐associated progeria. LMNAE262K is increasingly aggregated in the nucleus due to its intrinsic instability coupled with structural unfolding. Aggregates of LMNAE262K sequester various nuclear chaperones, proteasomal proteins, and DNA repair proteins to induce nuclear proteotoxicity and subsequent DNA damage.

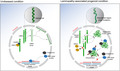

**Abbreviations**
DNA: deoxyribonucleic acid
HGPS: Hutchinson‐Gilford progeria syndrome
HSPA1A: heat shock protein family A (Hsp70) member 1A
LMNA: lamin A
MRE11: MRE11 homolog, double strand break repair nuclease
mRNA: messenger ribonucleic acid
NAT10: N‐acetyltransferase 10
nM: nanomolar
nm: nanometer
PCR: polymerase chain reaction
PSMD8: proteasome 26S subunit, non‐ATPase 8
ORF: open reading frame
RanBP2: RAN binding protein 2
RMSD: root mean square deviation
RMSF: root mean square fluctuation
SASA: solvent accessible surface area
S.D.: standard deviation
SUMO2: small ubiquitin like modifier 2
UBE2I: ubiquitin conjugating enzyme E2 I
UV: ultraviolet

## 1 INTRODUCTION

The imbalance of proteome in the nucleus of eukaryotic cells is orchestrated by the accumulation of misfolded proteins or suboptimal protein quality control systems, leading to a systemic failure of nuclear homeostasis (Enam et al., 2018). Mutant proteins could evade the surveillance mechanisms of quality control systems, and their overload in the nucleus in the form of aggregates could potentially lead to intrinsic proteotoxicity (Morimoto, 2008). Usually, nuclear protein aggregates form specific structures such as inclusion bodies, promyelocytic leukemia bodies, and speckles (Matera et al., 2009). In this context, not only nucleoplasmic proteins but also mutants of nuclear envelope proteins, such as lamins, could also cause nuclear pathogenicity in various rare diseases such as laminopathies. (Rankin & Ellard, 2006).

Laminopathies are a class of rare genetic disorders that are characterized by de novo heterozygous mutations in the lamin A (*LMNA*) gene (Kudlow et al., 2007). Clinically, many of the individuals with laminopathic disorders show several physiological symptoms of early aging, such as progeroid facial features, short stature with lower body weight, ectodermal tissue, and connective tissue defects etc. (Hennekam, 2006). Based on the mutation in *LMNA*, laminopathies are classified into typical and atypical forms (Hennekam, 2006). For example, Hutchinson–Gilford progeria syndrome (HGPS) is a typical laminopathy‐associated premature aging disorder. On the other hand, Malouf syndrome, mandibuloacral dysplasia and congenital muscular dystrophy are laminopathic disorders that show atypical features of early aging. Although the characteristics are similar, early‐onset laminopathies have more severe phenotypes than late‐onset laminopathies. In severe cases of laminopathies, lipoatrophy, cardiovascular problems (coronary occlusions), cerebrovascular occlusions, and stenosis are also observed (Hennekam, 2006).

Lamins (LMNA, LMNB, and LMNC) are a class of nuclear proteins that form the core of the meshwork of the lamina of nuclear envelope (Gruenbaum & Foisner, 2015). Structurally, lamin proteins form a filamentous network in the lamina that defines the proper shape and morphology of the nucleus (Gruenbaum & Foisner, 2015). In addition, they also function in maintaining the elasticity of the matrix, proper positioning of nuclear receptors, and mechanotransduction (Dubik & Mai, 2020). The structure of LMNA protein comprises two N‐terminal helical rod domains, typically forming a coiled‐coil region, and a C‐terminal globular beta‐sheet domain flanked by a long‐disordered region (Ahn et al., 2019). LMNA is synthesized as prelamin A, which is C‐terminally isoprenylated and farnesylated. A proteolytic cleavage of C‐terminal eighteen amino acids of post‐translationally modified prelamin A forms the mature LMNA (Simon & Wilson, 2013). The rod domains are amphipathic helices, while the C‐terminal region resembles a low‐complexity region enriched with serine, histidine, and glycine residues. The N195 residue is reported to be required for nuclear translocation of LMNA (Dechat et al., 2010). Besides its function of mechanically supporting the nuclear lamina, LMNA interacts with various proteins to regulate transcription during fibroblast proliferation and myoblast differentiation (Burke & Stewart, 2013).

Typical laminopathic cells show a deformed nuclear morphology with thick and lobular nuclear envelopes (Eriksson et al., 2003). Several of the mutations in *LMNA* result in this cellular phenotype. For example, a mutation in the 608th codon of *LMNA* creates a cryptic splice site that leads to a C‐terminally fifty amino acid truncated version of LMNA, called progerin (Eriksson et al., 2003). While progerin itself causes nuclear envelope disruption, its farnesylation exacerbates the condition (Yang et al., 2006). Some other truncation mutations of LMNA, such as Q432X (Yang et al., 2013), also lead to similar pathological features as progerin. A number of other studies have confirmed that the loss of binding of mutants of LMNA to nuclear envelope causes structural collapse of the nuclear lamina (Piekarowicz et al., 2017). Interestingly, most of these mutations are clustered in the C‐terminal globular domain of LMNA (Krimm et al., 2002), making this domain a mutational hotspot for laminopathic diseases. Although it is not known how the C‐terminal mutations render LMNA unable to tether to the nuclear envelope, an interplay of nesprin‐2 (Yang et al., 2013) and NAT10 (Larrieu et al., 2014) with LMNA may be a regulatory mechanism in this process. While many of the mutants of LMNA remain profusely distributed in the nucleoplasm (West et al., 2016), mutations in LMNA also lead to the protein's nucleoplasmic aggregation (Boudreau et al., 2012). Interestingly, mutations in both rod domains of LMNA can transform the protein into an aggregation‐prone entity, suggesting a common mechanism of aggregation of rod‐domain mutants of LMNA. Strikingly, a large number of disease‐associated mutations in LMNA involve substitutions of charged amino acids, suggesting that imbalance of charge distribution and hydrophobicity may lead to instability of LMNA under physiological conditions. However, a detailed mechanistic understanding and correlation between the mutations and the phenotypes of laminopathies are lacking.

In this study, we report a p.Glu262Lys (E262K) mutation in the second rod domain of LMNA in an individual with atypical progeroid manifestations. Mutation of the conserved glutamic acid to lysine in LMNA leads to destabilization of the protein by inducing a helix‐to‐disorder structural transition of the mutation region, thus forming a hydrophobic patch that promotes aggregation of the protein in aqueous environments. The E262K mutation also prevents SUMOylation of LMNA^E262K^ by preventing the binding of UBE2I to the mutant LMNA. Nucleoplasmic aggregates of LMNA^E262K^ not only resist degradation but also sequester nuclear chaperones, proteasomal proteins, and DNA repair proteins to deregulate nuclear proteostasis and DNA repair pathways.

## 2 RESULTS

### 2.1 P.Glu262Lys mutation of LMNA underscores the phenotypes of atypical progeroid condition

#### 2.1.1 Clinical report of the individual

**FIGURE 1:**
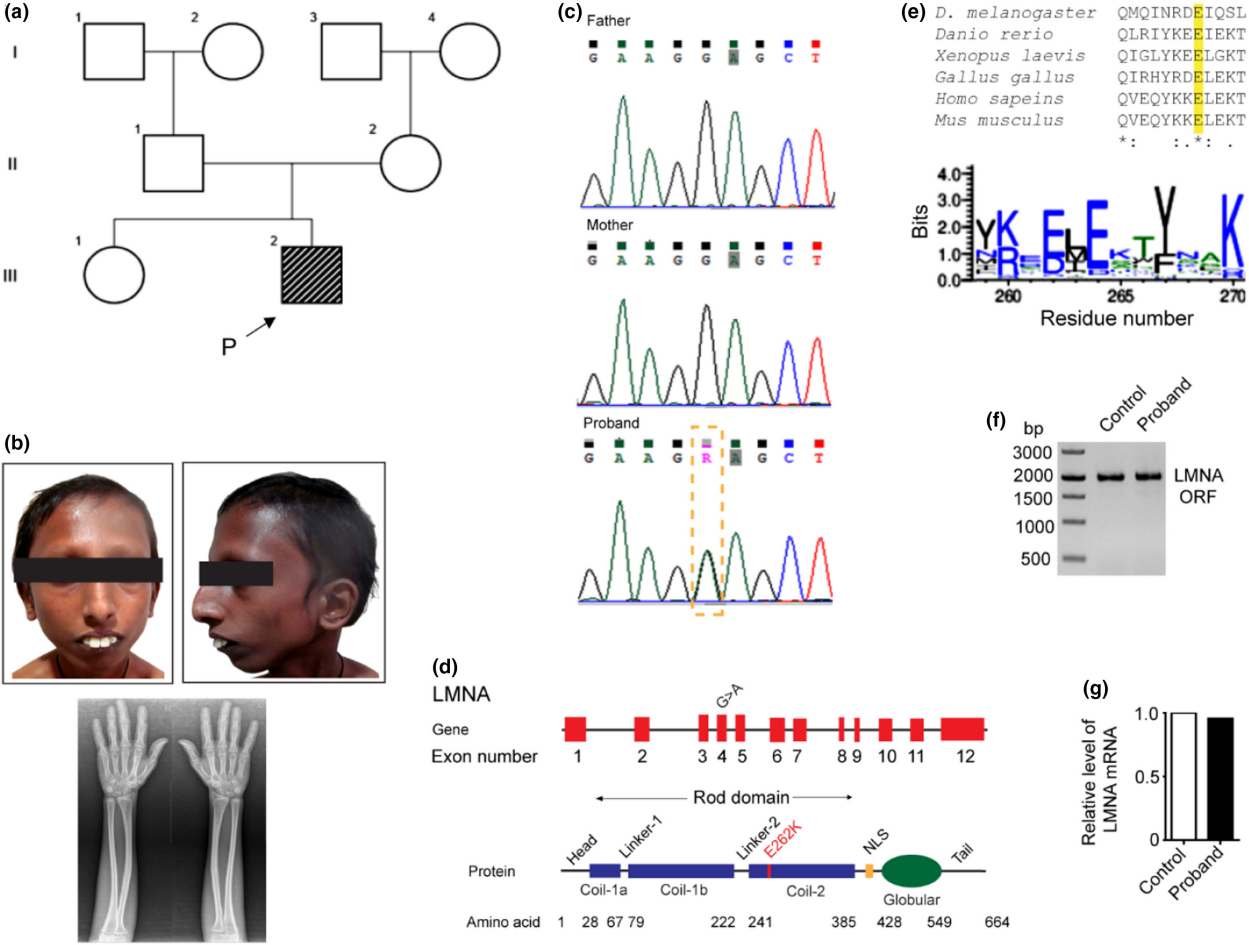
Whole exome sequencing identified a c.784G>A mutation in the exon‐4 of *LMNA* of the proband. (a) Pedigree of the proband over three generations. (b) Clinical photographs showing face and hand radiographs of the proband at sixteen years of age. (c) Sanger sequencing and segregation analysis of the mutation in *LMNA* of the proband and his parents, showing a de novo heterozygous base substitution. (d) Graphical representation of the mutated LMNA gene and protein showing the location of the mutation. (e and d) Multiple sequence alignment showing the conserved E262 residue of LMNA protein of different organisms. (f) Size of the PCR products of the reverse transcribed region of the open reading frame of the mature LMNA mRNAs of proband and control. (g) Relative abundance of LMNA mRNA, detected by RT‐PCR, in fibroblasts from proband and control

The proband is a second born male child of non‐consanguineous parents (Figure 1a). He had an uneventful antenatal period and born at term via normal vaginal delivery with a birth weight of 2.5 kg (−1.7 SD). His early growth and development were normal. Short stature, sparse hair, eyebrows and eyelashes, shallow orbits, narrow nasal bridge with broad nasal tip, craniofacial disproportion with micro‐retrognathia, and dental crowding were noted around sixteen years of age (Figure 1b). At this age, his weight was 21 kg (−2.6 SD), height was 141.5 cm (0.3 SD), and head circumference was 49 cm (−2.9 SD). While his fingernails were dark colored with longitudinal ridges, his hand radiographs were age appropriate without features of acroosteolysis (Figure 1b). The proband has high pitched voice, and he did not develop secondary sexual characters. Proband's echocardiography was unremarkable.

Exome sequencing was performed from the genomic DNA of the blood cells of the proband. A heterozygous substitution at g.156134949G>A (corresponding to c.784G>A in mature mRNA) was identified in exon 4 of the *LMNA* gene of proband (Figure 1c and Tables S1, S2, Figure S1). Sanger sequencing in his parents did not show this variation (Figure 1c), confirming the de novo status of the variant in the proband. Interestingly, the c.784G>A base substitution in the *LMNA* gene, resulting in a non‐conservative E262K missense mutation in the LMNA protein (Figure 1d). The E262K mutation had also been previously reported in an individual with atypical progeroid syndrome and lipodystrophy (Yukina et al., 2021). The in silico algorithms predicted this mutation as damaging and pathogenic (Figure S2).

The E262K mutation in LMNA occurs at the conserved sites (Figures 1e and S3). Therefore, this mutations would likely to generate unfavorable consequences on the structural and functional landscape of LMNA. Multiple sequence alignment of LMNA proteins of different organisms shows that the 262nd position of LMNA prefers acidic amino acids (Figure 1e). Substitution of this negatively charged amino acid with a basic amino acid, such as a lysine in LMNA^E262K^, would, as expected, affect the local stability of the region near the substitution.

Several of the laminopathy‐associated sequence variations in *LMNA* are known to induce alternative splicing by introducing cryptic splice sites in LMNA pre‐mRNA, resulting in the formation of different isoforms of LMNA protein (Rodriguez et al., 2009). PCR of the reverse transcribed product of the open reading frame (ORF) of LMNA mRNA from fibroblasts showed no difference in the LMNA‐ORF length of the proband compared to the control (Figure 1f). This observation showed that the c.784G>A base substitution did not generate a cryptic splice site in the mutant LMNA mRNA. Thus, LMNA^E262K^ protein predictably had the same length as the wild‐type LMNA protein. In addition, quantification of the mature mRNA of *LMNA* by qPCR showed no significant difference in the expression of the *LMNA* gene in the proband compared with control (Figure 1g). Therefore, the effect of the c.784G>A mutation in the *LMNA* gene on laminopathy‐associated progeroid manifestation is not related to the formation of alternative isoforms of LMNA nor to the differences in the expression of LMNA^E262K^ and LMNA. Alternatively, the early aging in the proband could be due to an aberrant structure–function association of LMNA^E262K^.

### 2.2 P.Glu262Lys mutation triggers aggregation of the second rod domain of LMNA^E262K^

**FIGURE 2:**
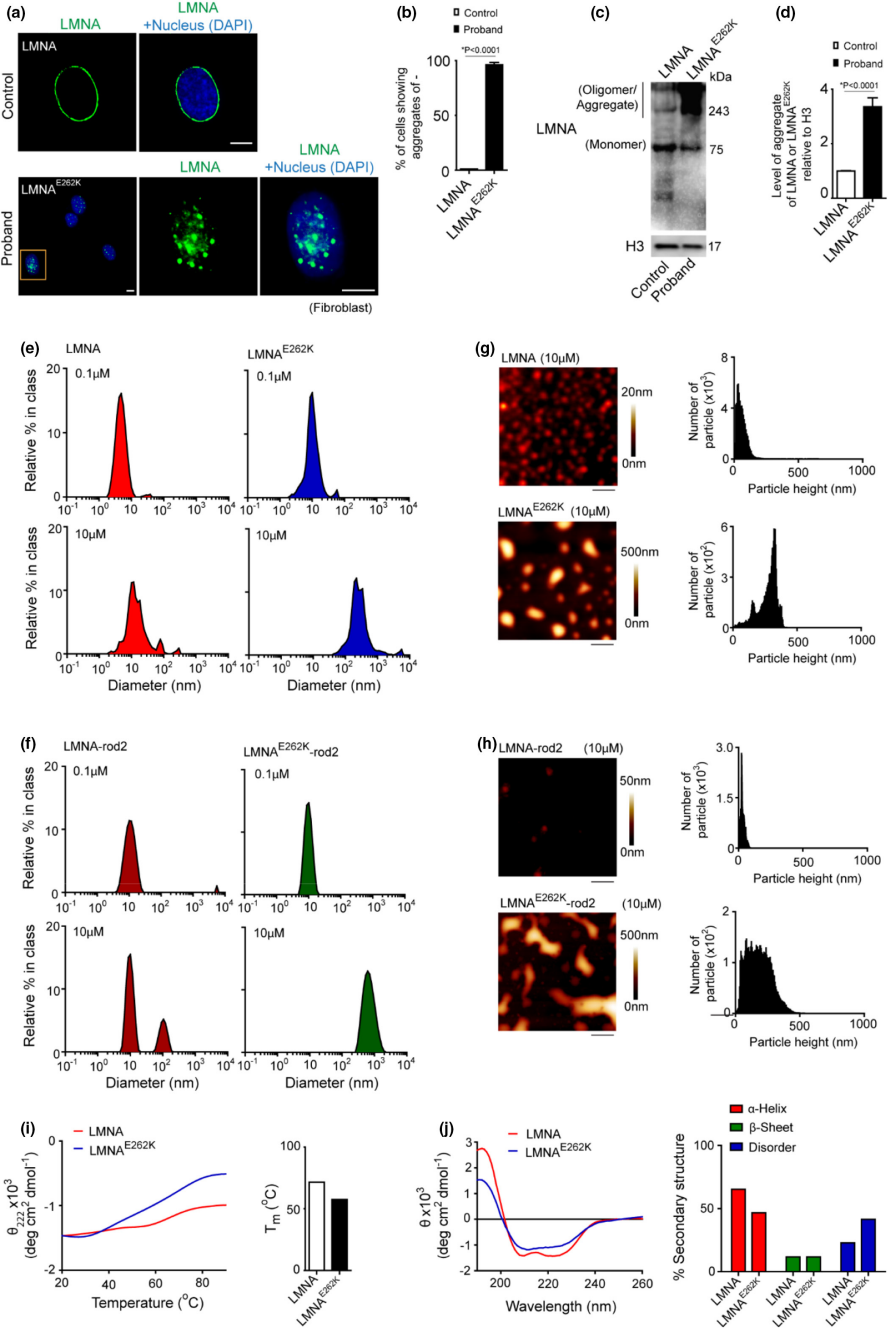
LMNA^E262K^ undergoes structural unfolding and concentration‐dependent aggregation. (a) Fibroblasts from proband and control (passage: 0, day: 5) were immunostained for LMNA. Representative images of confocal fluorescence microscopy show the distribution of LMNA in the nuclear envelope of control fibroblasts and the presence of nuclear aggregates of LMNA^E262K^ in the proband fibroblasts. (b) Quantification of the number of fibroblast cells showing nuclear aggregates of LMNA or LMNA^E262K^ in control and proband, respectively [100 cells analyzed]. (c) Representative immunoblot of LMNA and LMNA^E262K^ from whole cell lysate of control and proband fibroblasts (passage: 5, day: 2). Proteins of cell lysate were separated in 8% SDS‐PAGE. (d) Densitometric quantification of LMNA or LMNA^E262K^ aggregates relative to H3 (histone H3) of the immunoblot of 3C. (e) Dynamic light scattering of 0.1 and 10 μM LMNA and LMNA^E262K^ in aqueous environment. (f) Dynamic light scattering of 0.1 and 10 μM LMNA‐rod2 and LMNAE262K‐rod2 (226–387 amino acid region) in aqueous environment. (g) Ex situ atomic force microscopy images of 10 μM LMNA and LMNA^E262K^ show smaller oligomers of LMNA and phase‐separated liquid droplets of LMNA^E262K^. Scale bars show 2 μm. Height distribution of particles in AFM images shown. (h) Ex situ atomic force microscopy images of 10 μM LMNA‐rod2 and LMNA^E262K^‐rod2 show large globular aggregates of LMNA^E262K^‐rod2. The scale bars show 2 μm. The height distribution of LMNA‐rod2 and LMNA^E262K^‐rod2 particles in the AFM images is shown. (i) 1 μM LMNA or LMNA^E262K^ were heated slowly (1°C/min) and continuously from 20 to 90°C in aqueous buffer. The changes in molar residue ellipticity (θ_222_) of LMNA and LMNA^E262K^ at a light wavelength of λ = 222 nm were recorded by CD spectroscopy. The melting temperatures (T_m_) of LMNA and LMNA^E262K^ are shown. (j) The change in molar residue ellipticity (θ) of 1 μM LMNA and LMNA^E262K^ was recorded in the far UV wavelength range (190–260 nm). The CD spectra of LMNA and LMNA^E262K^ were deconvoluted using the CDSSTR program to show the different proportions of secondary structures (α‐helix, β‐sheet, and disorder) in the proteins. Quantifications are shown as mean ± SD; *p* values are indicated. H3 is the loading control in the immunoblots. Scale bar in confocal microscopy images: 5 μm. Microscopy and immunoblot data are representative of at least three independent experiments

We examined the expression and localization of LMNA and LMNA^E262K^ in the control and proband fibroblasts. Strikingly, mutant LMNA formed nuclear aggregates in a significant number of fibroblasts of proband and showed a loss of its localization in the nuclear envelope (Figure 2a,b). In addition, LMNA^E262K^ also showed a higher accumulation in cell (Figure 2c,d). LMNA^E262K^ showed SDS insoluble aggregates which were observed as the higher molecular weight complexes in the immunoblot. Since the transcript level of LMNA^E262K^ in the proband was not significantly different from the LMNA transcript in the control, the higher level of LMNA^E262K^ in the proband compared to LMNA in the control may be due to decreased degradation of the former protein in the proband.

LMNA contains two N‐terminal helical rod domains and a C‐terminal globular domain of beta‐sheets. Although the globular domain is a hotspot for laminopathy‐associated mutations, structural destabilization of LMNA is also driven by mutations in the rod domains of the protein. There is strong evidence that multiple mutations in the rod domains cause aggregation of LMNA (Boudreau et al., 2012; Piekarowicz et al., 2017), although the mechanism of this phenomenon is not clear. Having established that LMNA^E262K^ aggregates in proband fibroblasts, we speculated that the E262K mutation contributes critically to the aggregation of LMNA, probably by modulating the stability of the second rod domain of the protein. To test this hypothesis, we purified bacterially expressed recombinant LMNA, LMNA^E262K^, and their individual second rod domains—LMNA‐rod2 and LMNA^E262K^‐rod2 (residues 226–387), and tested their ability to aggregate. Both LMNA^E262K^ and LMNA^E262K^‐rod2 formed aggregates in a concentration‐dependent manner, whereas LMNA and LMNA‐rod2 did not form aggregates even at higher concentrations (Figure 2e–h). Aggregates of LMNA^E262K^ and LMNA^E262K^‐rod2 formed in solution were probed by dynamic light scattering. At a higher concentration (10 μM), LMNA^E262K^ and LMNA^E262K^‐rod2 formed soluble aggregates with the most abundant particulate diameters in the range of 192–342 nm and 458–1106 nm, respectively (Figure 2e,f). The formation of soluble aggregates of LMNA^E262K^ and LMNA^E262K^‐rod2 was insensitive to salt concentrations, suggesting that aggregation of the rod2 domain of LMNA^E262K^ is driven by hydrophobic interactions. Ex situ measurements of protein particles' height distribution by atomic force microscopy also revealed large aggregates of LMNA^E262K^ and LMNA^E262K^‐rod2 at higher concentrations (Figure 2g,h). The aggregates of LMNA^E262K^ and LMNA^E262K^‐rod2 were not fibrillar but appeared as liquid droplets, suggesting that the aggregates were probably formed by phase separation in a mechanism similar to that of other aggregation‐prone proteins such as MAPT (also known as tau; Kanaan et al., 2020). On the other hand, LMNA did not form large particulates in solution. At higher concentration (10 μM), LMNA formed most abundant particulates with a diameter of 7.5–92 nm, while LMNA‐rod2 showed two distinct particle populations with diameters ranging from 6.5–15 to 68–164 nm (Figure 2e,f). In atomic force microscopy, LMNA and LMNA‐rod2 did not show large droplets but smaller oligomeric organization (Figure 2g,h). At lower concentration (100 nM), LMNA and LMNA‐rod2 showed particles with diameter of <10 nm. These results indicate that the second rod domain of LMNA is intrinsically prone to oligomerization, possibly due to its coiled‐coil structural form. However, the E262K mutation transforms the rod2 domain into a structure which is more prone to aggregation.

To understand whether the E262K mutation alters the structural properties of LMNA^E262K^ compared to LMNA, we analyzed the secondary structure components of LMNA and LMNA^E262K^. Slow thermal denaturation during circular dichroism spectroscopy at 222 nm light wavelength revealed faster melting of LMNA^E262K^ than wild‐type LMNA (Figure 2i), suggesting that LMNA^E262K^ contains a lower proportion of secondary structures than wild‐type LMNA. Furthermore, scanning at far UV wavelengths in circular dichroism spectroscopy clearly confirmed a reduction in helical structures and an increase in the disordered region of LMNA^E262K^ compared with wild‐type LMNA (Figure 2j).

Thus, aggregation of LMNA^E262K^ not only required the second rod domain but is also coupled with unfolding of one or more regions of the protein.

### 2.3 Unfolding of the mutation‐harboring region of LMNA^E262K^ generates an aggregation‐prone hydrophobic patch

**FIGURE 3:**
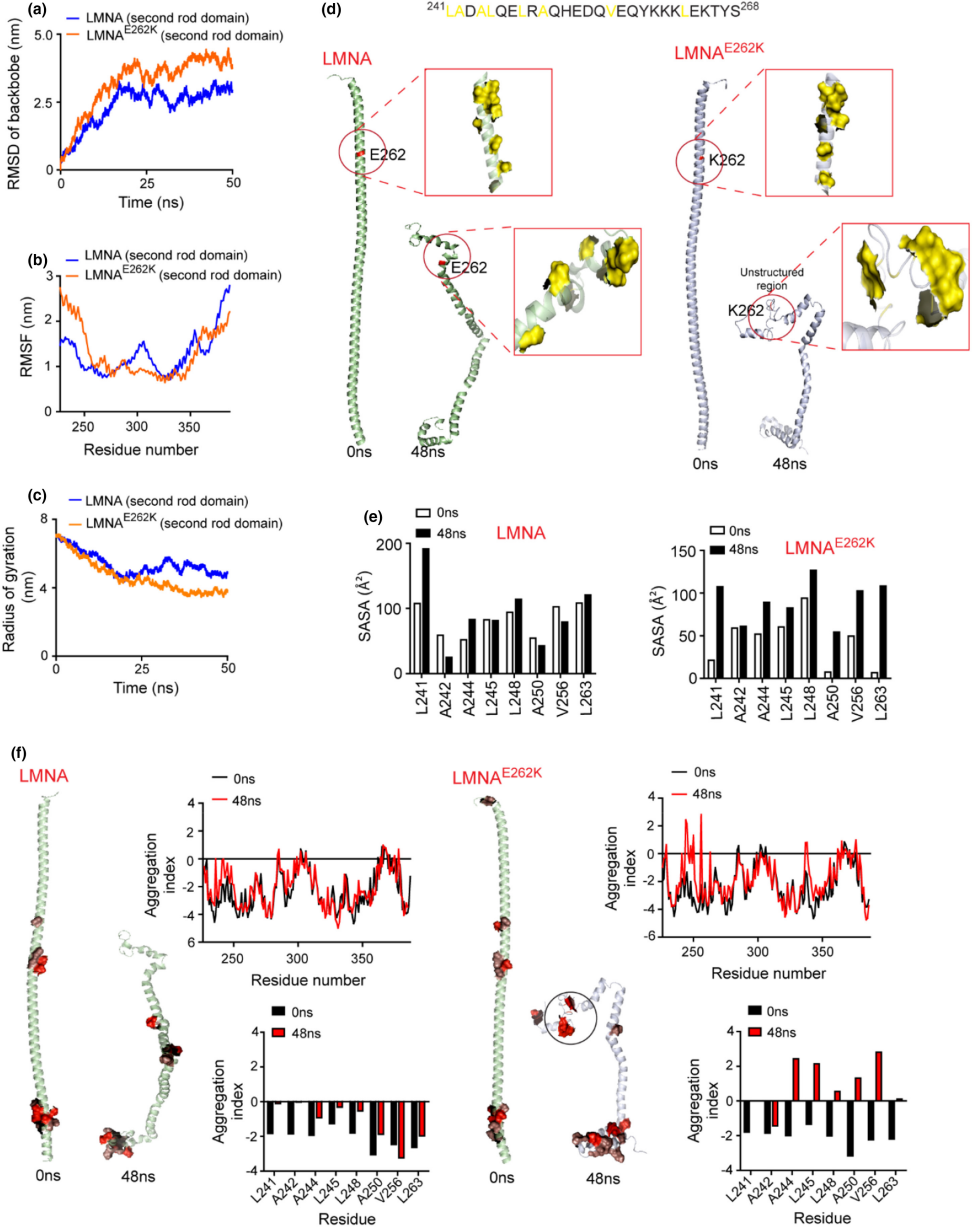
Structural unfolding of the mutational region in the rod2 domain of LMNA^E262K^ generates two aggregation‐prone hydrophobic patches. (a) Root mean square deviation (RMSD) values of the backbones of the rod2 domains (226–387 amino acids) of LMNA and LMNA^E262K^ during molecular dynamics simulation showing higher RMSD values (greater instability) of the backbone of LMNA^E262K^‐rod2. (b) Root mean square fluctuation (RMSF) values of the residues of LMNA‐rod2 and LMNA^E262K^‐rod2 over the simulation period, showing higher fluctuation (instability) of the N‐terminal region upstream of the E262K mutation of LMNA^E262K^‐rod2. (c) Radius of gyration of LMNA‐rod2 and LMNA^E262K^‐rod2 during the simulation period. The lower values of the gyration radius of LMNA^E262K^‐rod2 in the later simulation period represent a more rigid structure of this protein. (d) Snapshots of images of LMNA‐rod2 and LMNA^E262K^‐rod2 at 0 and 48 ns of simulation showing the scattered or continuous‐clustered distribution of E262/K262‐upstream hydrophobic residues of LMNA‐rod2 and LMNA^E262K^‐rod2, respectively, over time. (e) Quantification of solvent accessible surface area (SASA) of specific hydrophobic residues of LMNA‐rod2 and LMNA^E262K^‐rod2 at 0 and 48 ns simulation time. (f) Predictive aggregation index of residues in LMNA‐rod2 and LMNA^E262K^‐rod2 at 0 and 48 ns simulation time showing the aggregation propensity of the specific hydrophobic residues near the E262K mutation in the 48 ns structure of LMNA^E262K^‐rod2

To understand how unfolding relates to the aggregation of LMNA^E262K^‐rod2 in a real‐time setting, we performed unconstrained molecular dynamics simulations of the LMNA‐rod2 and LMNA^E262K^‐rod2 structures. The simulation data showed that the LMNA^E262K^‐rod2 is more unstable than the LMNA‐rod2. The higher root mean square deviation (RMSD) values of the backbone of LMNA^E262K^‐rod2 indicated greater instability of the protein compared to LMNA‐rod2 (Figure 3a). The higher root mean square fluctuation (RMSF) values of K262 and the neighboring residues in LMNA^E262K^‐rod2 compared with E262 and the neighboring residues of LMNA‐rod2 (Figure 3b) indicated specific instability of the N‐terminal region of LMNA^E262K^‐rod2. Accordingly, the overall structure of LMNA^E262K^‐rod2 became more rigid than the structure of LMNA over time, as evidenced by a smaller radius of gyration (R_g_) of LMNA^E262K^‐rod2 than LMNA‐rod2 (Figure 3c).

The K262 and its upstream helical regions (residues 242–268) in LMNA^E262K^‐rod2 were rapidly unfolded into an unstructured region (Figures 3d and S4), and the nascent unfolded region reorganized such that several of the hydrophobic residues (A242, A244, L245, L248, A250, V256, and L263) in this region were brought closer together to form a topology of two continuous hydrophobic patches (Figure 3d). In contrast, E262 and the upstream region remained as an intact helix (Figure 3d), and the hydrophobic residues in the 242–268 amino acid region of LMNA‐rod2 did not undergo proximal positioning (Figure 3d). Moreover, the solvent‐accessible surface area (SASA) of many hydrophobic residues in the two patches of LMNA^E262K^‐rod2 was higher than that SASA of the corresponding hydrophobic residues of LMNA‐rod2 (Figure 3e). Since the exposure of the hydrophobic segments to the aqueous environment is thermodynamically unfavorable, the two solvent‐exposed hydrophobic patches of LMNA^E262K^‐rod2 exposed to the solvent also contributed to the decrease in the energetic stability of the protein.

Analysis of the equilibrium data revealed the aggregation index of the different regions of LMNA‐rod2 and LMNA^E262K^‐rod2 over the simulation period. While both structures contained an aggregation‐prone region in the C‐terminal side of the rod domain (Figure 3f), the temporal unfolding‐coupled clustering of the nonpolar residues in the two hydrophobic patches near the mutation of LMNA^E262K^‐rod2 also resulted in an aggregation propensity in these residues (Figure 3f). The extended hydrophobicity of the patches appeared to correlate with the aggregation properties of the mutation region of LMNA^E262K^‐rod2. In contrast, the hydrophobic residues near E262 of LMNA‐rod2 did not induce aggregation properties (Figure 3f). Because the hydrophobic residues near E262 of LMNA‐rod2 were scattered and surrounded by polar and charged residues, they were more in equilibrium with water and did not have much aggregation potential.

Taken together, these results indicate that local unfolding of helical structures near the E262K mutation of LMNA^E262K^ facilitates juxta‐positioning of hydrophobic residues that act as aggregation‐prone patches in aqueous environments.

### 2.4 P.Glu262Lys mutation inhibits SUMOylation of LMNA^E262K^ by preventing binding of UBE2I to LMNA^E262K^

**FIGURE 4:**
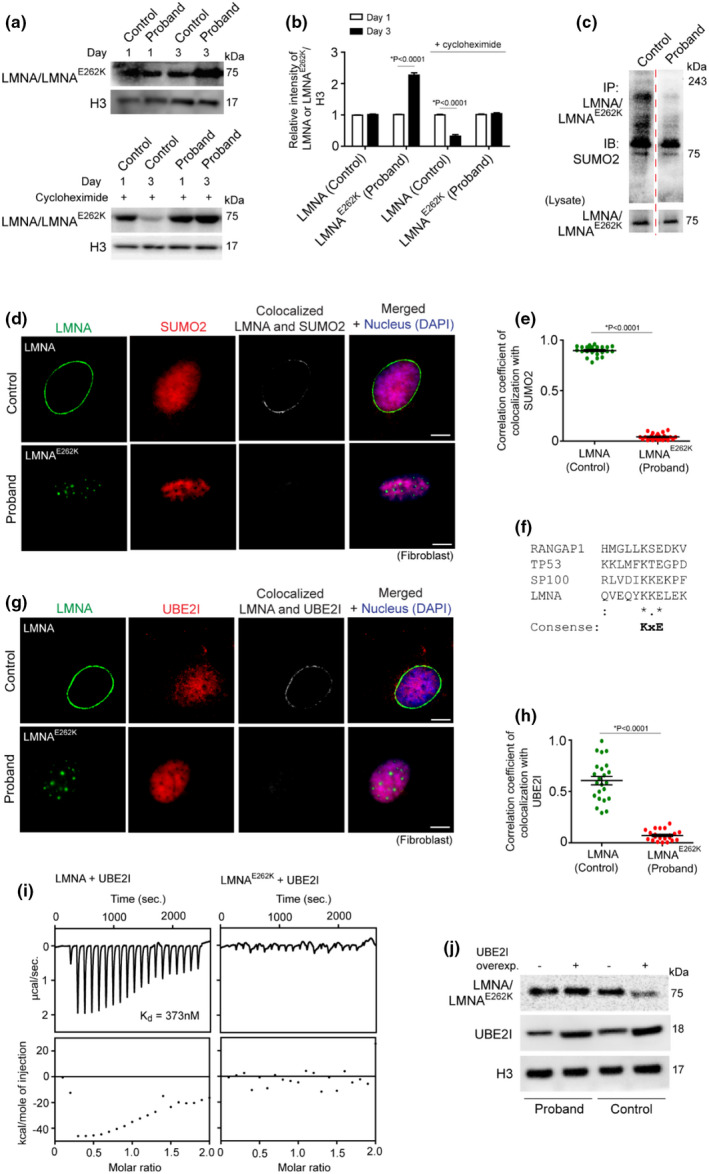
Reduced SUMOylation of LMNA^E262K^ due to loss of binding of UBE2I to LMNA^E262K^ results in nuclear accumulation of mutant LMNA in proband fibroblasts. (a) Top: Representative immunoblot of LMNA and LMNA^E262K^ from whole cell lysate of control and proband fibroblasts (passage: 0, day: 1 and 3 after revival of cells). Bottom: Representative immunoblot LMNA and LMNA^E262K^ from whole cell lysate of 100 nM cycloheximide‐treated control and proband fibroblasts (passage: 0, day: 1 and 3 after revival of cells). Proteins of cell lysate were separated in 8% SDS‐PAGE. (b) Densitometric quantification of LMNA or LMNA^E262K^ level relative to H3 from immunoblot of 4A. (c) Representative immunoblots of SUMO2 and LMNA from the denaturing (with 1% SDS) immune complexes of LMNA and LMNA^E262K^ from lysate of proband and control fibroblasts. The higher molecular weight bands represent multi‐monoSUMOylated LMNA/LMNA^E262K^. Proteins of immunoprecipitation fraction were separated in 8% SDS‐PAGE. (d) Representative confocal immunofluorescence microscopy images of LMNA, LMNA^E262K^ and SUMO2 in control and proband fibroblasts. Colocalized pixels of LMNA/LMNA^E262K^ and SUMO2 are shown. (e) Quantitative estimation of correlation coefficients of colocalization of SUMO2 with LMNA and LMNA^E262K^. Colocalization coefficients are analyzed by measuring the pixel intensities of LMNA/LMNA^E262K^ and SUMO2 at nuclear envelope [25 cells analyzed]. (f) Multiple sequence alignment of UBE2I‐binding regions of various proteins, including the putative UBE2I‐binding region ^259^YKKE^262^ in wild‐type LMNA. (g) Representative confocal immunofluorescence microscopy images of LMNA, LMNA^E262K^ and UBE2I in control and proband fibroblasts. Colocalized pixels of LMNA/LMNA^E262K^ and UBE2I are shown. (h) Quantitative estimation of correlation coefficients of colocalization of UBE2I with LMNA or LMNA^E262K^. Colocalization coefficients are analyzed by measuring the pixel intensities of LMNA/LMNA^E262K^ and UBE2I at nuclear envelope [25 cells analyzed]. (i) Quantitative analysis of the interaction of recombinant UBE2I with LMNA and LMNA^E262K^ determined by isothermal titration calorimetry assays; the value of the dissociation constant (K_d_) of the binding of UBE2I to LMNA is indicated. (j) Control and proband fibroblasts (passage: 3) were untransfected or transfected with a UBE2I‐overexpressing clone. Representative immunoblots of LMNA or LMNA^E262K^ and UBE2I from the lysate of the above cells. Proteins of cell lysate were separated in 8% SDS‐PAGE

Having established the mechanism of aggregation of LMNA^E262K^, we sought to determine whether there were differences in clearance of LMNA^E262K^ in proband fibroblasts compared to LMNA in control fibroblasts and whether modulation of posttranslational modifications of LMNA^E262K^ affected its differential degradation. Immunoblotting against LMNA from nuclear lysate of proband and control fibroblasts clearly showed higher accumulation of LMNA^E262K^ in proband fibroblasts (Figure 4a,b). Because transcriptional expression of the LMNA gene was not significantly different in proband compared with control, the data suggest that LMNA^E262K^ protein is more resistant to degradation than LMNA. To further validate the lower intracellular degradation and higher half‐life of LMNA^E262K^ compared to LMNA, we checked the level of wild‐type and mutant LMNA in control and proband fibroblasts that were treated cycloheximide (translation inhibitor). While the level of LMNA temporally decreased in the cycloheximide‐treated control fibroblasts (Figure 4a,b), the level of LMNA^E262K^ did not reduce significantly over time in cycloheximide‐treated proband fibroblasts (Figure 4a,b), indicating that LMNA^E262K^ is more resistant to degradation in cellular system.

SUMOylation regulates the LMNA degradation potential under various physiological and stressed conditions (Zhang & Sarge, 2008). SUMOylation of nuclear LMNA during DNA damage and replication stress facilitates nucleophagy (Li et al., 2019), whereas cardiomyopathy‐related mutants of LMNA, such as K201R, E203G, and E203K, exhibit loss of SUMOylation (Zhang & Sarge, 2008). Several lysine residues of LMNA are targets of SUMOylation by SUMO2 (Hendriks et al., 2017), and we tested whether the E262K mutation can abolish SUMOylation of the lysine residues of LMNA^E262K^. Denaturing immunoprecipitation of LMNA and LMNA^E262K^ from the nuclear lysates of fibroblasts from Proband and control, followed by immunoblotting against SUMO2 and LMNA, showed lower SUMOylation of LMNA^E262K^ of proband compared with LMNA of control (Figure 4c). Similarly, LMNA^E262K^ of proband did not show as strong colocalization with SUMO2 in the nucleus as colocalization of SUMO2 with LMNA of control (Figure 4d,e). The lack of SUMOylation of aggregated LMNA mutant has been reported previously (Zhang & Sarge, 2008), and our finding of the loss of SUMOylated LMNA^E262K^ in laminopathy‐associated progeroid condition supports the idea that aggregated nuclear LMNA restricts its SUMOylation by one or more mechanisms.

UBE2I is a ubiquitous E2 ligase known to SUMOylate LMNA (Li et al., 2019). To determine the possible reason for the lack of SUMOylation of LMNA^E262K^, we focused on the effects of the E262K mutation on the binding of UBE2I to LMNA^E262K^. Sequence analysis revealed a consensus UBE2I binding site (ΨKxE, Ψ is a large hydrophobic amino acid, (Bernier‐Villamor et al., 2002)) at ^259^YKKE^262^ of LMNA (Figure 4f), which is also the region mutated in LMNA^E262K^. Because the E262K mutation disrupts the consensus binding site of UBE2I in LMNA^E262K^ (the sequence is ^259^YKKK^262^ in LMNA^E262K^), we suspected that UBE2I would not bind to LMNA^E262K^. Indeed, UBE2I showed significantly less colocalization with LMNA^E262K^ aggregates in proband fibroblasts compared with its colocalization with LMNA in control fibroblasts (Figure 4g,h). Since UBE2I remains in a multisubunit complex of RanBP2/RanGAP1‐ SUMO/UBE2I (Werner et al., 2012), we tested whether LMNA interacts directly with UBE2I or via other subunits of the E3 ligase complex. Isothermal titration calorimetry showed a high binding affinity of recombinant UBE2I to LMNA but not to LMNA^E262K^ (Figure 4i), indicating a direct association of UBE2I with the ^259^YKKE^262^ region of LMNA and an inability to interact with the ^259^YKKK^262^ of LMNA^E262K^.

Based on the above observation, we checked whether the absence of SUMOylation of LMNA^E262K^ mediated by UBE2I was responsible for its decreased degradation in proband. Overexpression of UBE2I in control fibroblasts decreased the nuclear concentration of LMNA, but the same effect was not observed in UBE2I‐overexpressed proband fibroblasts (Figure 4j), proving that the increased accumulation of LMNA^E262K^ and its aggregates in proband fibroblasts is due to the lack of SUMOylation of LMNA^E262K^ due to the nonbinding of UBE2I to LMNA^E262K^.

### 2.5 Aggregates of LMNA^E262K^ disrupt nuclear proteostasis

**FIGURE 5:**
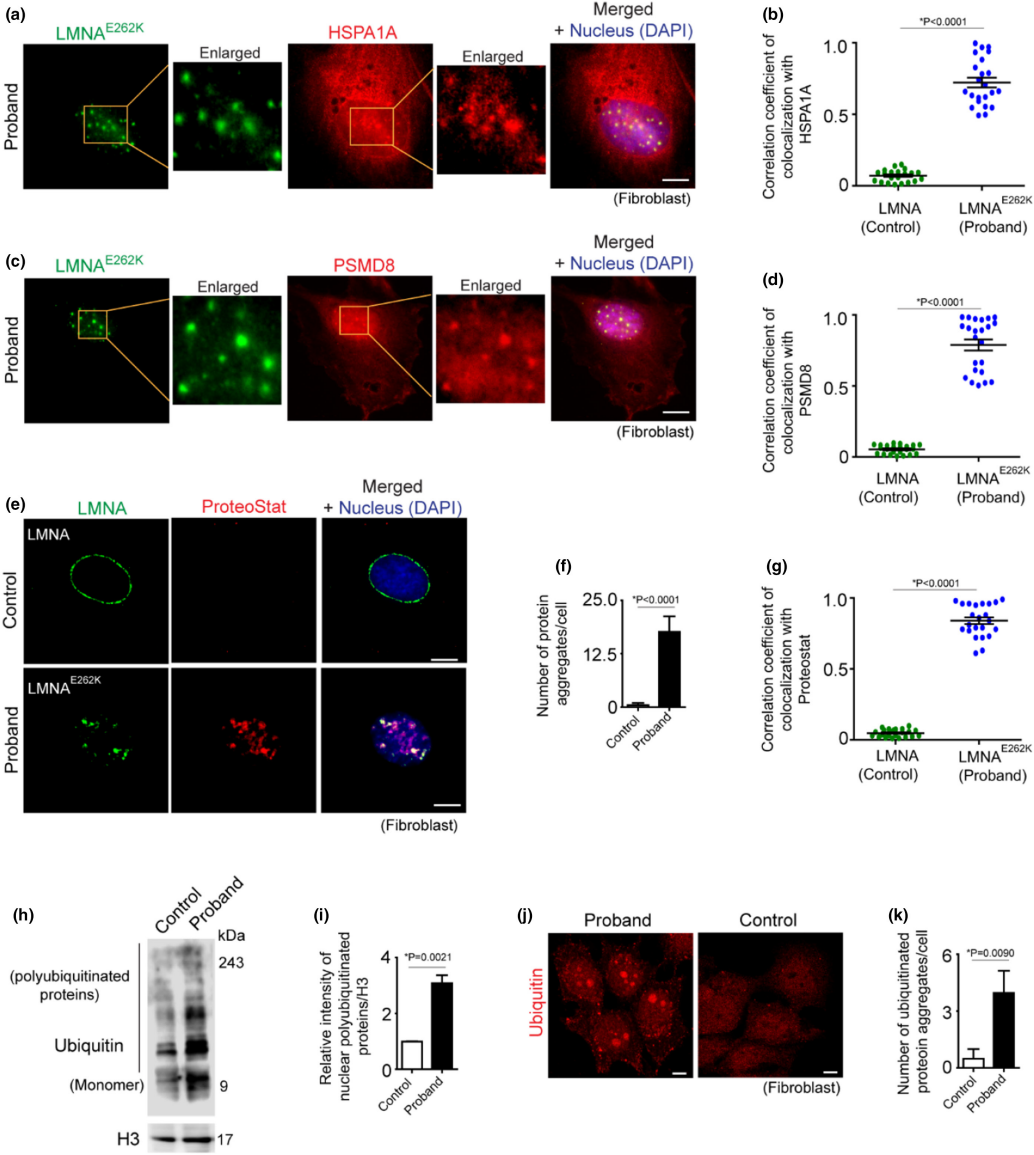
Aggregates of LMNAE262K deregulate nuclear proteostasis. (a) Representative confocal immunofluorescence microscopy images of LMNAE262K and HSPA1A in proband fibroblasts showing sequestration of HSPA1A by aggregates of LMNAE262K. (b) Quantitative estimation of correlation coefficients of colocalization of HSPA1A with LMNAE262K. [25 cells analyzed] (c) representative confocal immunofluorescence microscopy images of LMNAE262K and PSMD8 in proband fibroblasts showing sequestration of nuclear proteasomes by the aggregates of LMNAE262K. (d) Quantitative estimation of correlation coefficients of colocalization of PSMD8 with LMNAE262K [25 cells analyzed]. (e) Representative confocal fluorescence microscopy images of LMNA, LMNAE262K, and Proteostat. (f) Quantification of Proteostat‐positive nuclear protein aggregates in control and proband fibroblasts [100 cells analyzed]. (g) Quantitative estimation of correlation coefficients of Proteostat colocalization with LMNAE262K [25 cells analyzed]. (h) Representative immunoblot of ubiquitin from nuclear lysate of fibroblasts from proband and control. Proteins of nuclear lysate were separated in 8% SDS‐PAGE. (i) Densitometric quantification of ubiquitin level relative to H3 of immunoblot of 5H. (j) Representative confocal immunofluorescence microscopy images of ubiquitin in control and proband fibroblasts showing accumulation of ubiquitinated protein aggregates in the nucleus of proband fibroblasts. (k) Quantification of the number of ubiquitinated protein aggregates in the nucleus of control and proband fibroblasts [100 cells analyzed]. Quantifications are shown as mean ± SD; *p* values are indicated. H3 is the loading control in the immunoblots. Scale bar in confocal microscopy images: 5 μm. Microscopy and immunoblot data are representative of at least three independent experiments

To understand the mechanisms linking aggregation of LMNA^E262K^ to proteotoxicity, we investigated whether accumulation of LMNA^E262K^ aggregates globally disrupt nuclear proteostasis. Because phase‐separated aggregates nonspecifically sequester other proteins (Yang & Hu, 2016), LMNA^E262K^ aggregates could attract and sequester essential proteostasis‐maintaining proteins. Based on co‐immunostaining of LMNA and HSPA1A (chaperone protein HSP70, member 1A) and LMNA and PSMD8 (proteasomal protein), followed by fluorescence analysis in proband fibroblasts, significant colocalization of HSPA1A and PSMD8 with nuclear aggregates of LMNA^E262K^ was evident (Figure 5a–d), implying sequestration of chaperones and proteasomal proteins by LMNA^E262K^ aggregates. We found that sequestration of chaperones such as HSPA1A by LMNA^E262K^ aggregates promoted the formation of nuclear aggregates, as evidenced by positive staining of these aggregates with Proteostat dye in the nucleus of proband fibroblasts (Figure 5e,f), whereas the nucleus of control fibroblasts did not show a significant amount of protein aggregates (Figure 5e,f). Interestingly, Proteostat not only stained LMNA^E262K^ aggregates, but there was also an abundance of non‐LMNAE262K aggregates in the nucleus (Figure 5g). These data suggest that sequestration of HSPA1A (and probably other nuclear chaperones) by LMNA^E262K^ aggregates reduced the pool of active chaperones in the nucleoplasm, a phenomenon that correlated with a global failure of nuclear proteostasis, leading to the formation of aggregates of various proteins in the nucleus. Using ubiquitin staining, we found that LMNA^E262K^‐induced proteotoxicity not only formed nuclear protein aggregates, but that these aggregates were progressively ubiquitinated (Figure 5h,i). However, because of possible inactivation of proteasomes sequestered by LMNA^E262K^, the ubiquitinated proteins were not optimally degraded, and their successive accumulation led to reorganization of the ubiquitinated protein aggregates in the form of large spheres (Figure 5j,k). A combination of the above data is consistent with the phenotypes of nuclear proteotoxicity and suggests an active role of LMNA^E262K^ in triggering nuclear stress through the formation of heterogeneous protein aggregates.

### 2.6 Nuclear proteostasis imbalance by LMNA^E262K^ aggregates causes accumulation of DNA damage and senescence in proband fibroblasts

**FIGURE 6:**
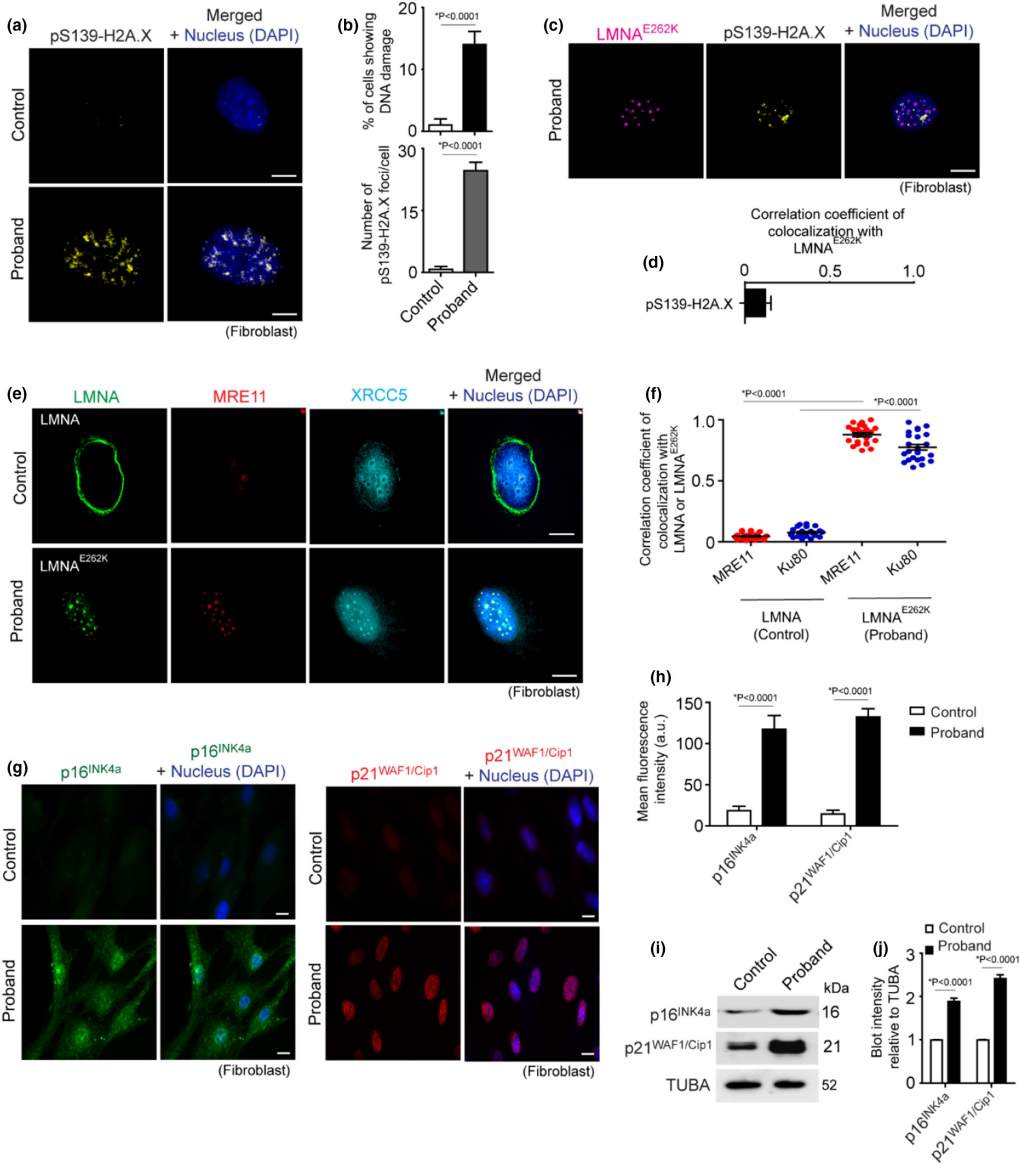
Sequestration of DNA damage repair proteins by LMNA^E262K^ aggregates leads to DNA damage accumulation in proband fibroblasts. (a) Representative confocal immunofluorescence microscopy images of pS139‐H2A.X‐positive damaged DNA foci in control and proband fibroblasts. (b) Top: Quantification of the percentage of cells with significantly damaged DNA foci in control and proband fibroblasts [100 cells analyzed]. Bottom: Quantification of pS139‐H2A.X‐positive damaged DNA foci in control and proband fibroblasts [100 cells analyzed]. (c) Representative confocal immunofluorescence microscopy images of LMNA^E262K^ and pS139‐H2A.X proband fibroblasts. (d) Quantitative estimation of correlation coefficients of colocalization of pS139‐H2A.X with LMNA^E262K^ [100 cells analyzed]. (e) Representative confocal immunofluorescence microscopy images of LMNA, LMNA^E262K^, MRE11, and XRCC5 in control and proband fibroblasts. (f) Quantitative estimation of the correlation coefficients of colocalization of MRE11 and XRCC5 with LMNA and LMNA^E262K^ [25 cells analyzed]. (g) Representative confocal immunofluorescence microscopy images of p16^INK4a^ and p21^WAF1/Cip1^ in control and proband fibroblasts. (h) Quantification of mean fluorescence intensity of p16^INK4a^ and p21^WAF1/Cip1^ in control and proband fibroblasts [100 cells analyzed]. (i) Representative immunoblot of p16^INK4a^ and p21^WAF1/Cip1^ from lysate of control and proband fibroblasts. Proteins of lysate were separated in 8% SDS‐PAGE. (j) Densitometric quantification of p16^INK4a^ and p21^WAF1/Cip1^ level relative to H3 of immunoblot of 6I. Quantifications are shown as mean ± SD; *p* values are indicated. Scale bar in confocal microscopy images: 5 μm. Microscopy and immunoblot data are representative of at least three independent experiments

Aggregation of proteins in the nucleus has emerged as one of the major DNA‐damaging stressors in nucleopathies (Gruenbaum & Foisner, 2015). Immunocytochemistry against the DNA damage marker phosphor‐serine‐139‐H2A.X (pS15‐H2A.X, also known as γ‐H2A.X) showed a higher number of γ‐H2A.X‐positive foci in a significant number of proband fibroblasts compared with control fibroblasts (Figure 6a,b), representing a higher level of DNA damage in the proband cells. However, the damaged LMNA^E262K^ aggregates did not exhibit coaccumulation of damaged DNA foci, as indicated by the very low colocalization of LMNA^E262K^ with γ‐H2A.X (Figure 6c,d). The lack of physical association of LMNA^E262K^ with damaged DNA sites suggests a passive mechanism that blocks DNA repair by LMNA^E262K^ aggregates.

Previous studies have shown that proteomic stress perturbs DNA repair pathways and associated signaling mechanisms (McAdam et al., 2016; Squier, 2001). As with HSPA1A and PSMD8, LMNA^E262K^ aggregates were observed to sequester many of the essential DNA damage repair proteins such as MRE11 and XRCC5 (also known as KU80; Figure 6e,f). While these DNA repair proteins were diffusely distributed in the nucleus of control fibroblasts, they were highly enriched in LMNA^E262K^ aggregates (Figure 6e,f). Therefore, it was evident that proteotoxic stress in nucleus modulated the decreased DNA repair process in proband fibroblasts.

Progeroid cells and DNA damage are known to trigger cellular senescence (d'Adda di Fagagna, 2008; Wheaton et al., 2017). Loss of nuclear lamins is also observed in aging cells undergoing senescence (Matias et al., 2022). Therefore, we further investigated the extent to which loss of LMNAE262K from the nuclear envelope and DNA damage induce senescence in the proband fibroblasts. Because increased expression of p16^INK4a^ and p21^WAF1/Cip1^ are hallmarks of senescent cells (Kohli et al., 2021; Matias et al., 2022), we checked the levels of these proteins in control and proband fibroblasts. Compared with the control fibroblasts, the proband fibroblasts showed significantly increased expression of p16^INK4a^ and p21^WAF1/Cip1^ (Figure 6g–j), indicating that the proband fibroblasts were undergoing senescence.

From these observations, we concluded that sequestration of DNA damage repair proteins by LMNA^E262K^ aggregates induces critical genotoxicity, effectively leading to deregulation of DNA damage repair pathways and cellular senescence in proband cells.

## 3 DISCUSSION

Premature aging is a class of developmental disorders that are characterized by genetic mutations and hallmarked by proteomic imbalance in the cell (Morimoto & Cuervo, 2014). The shift in proteostasis during normal aging overloads the cellular protein quality control system with nonfunctional and toxic protein forms, such as misfolded and aggregated proteins, resulting in proteotoxicity (David, 2012). While in normal aging, the effectiveness of chaperones and proteolytic mechanisms is gradually reduced by various molecular mechanisms, in premature aging diseases, particularly in various types of progeria and neurodevelopmental disorders, the components of the protein quality control system are overwhelmed by the accumulation of one or more mutant protein aggregates, thereby modulating organellar homeostasis and cell survival signaling pathways (Dreesen, 2020). However, whereas normal age‐related proteotoxicity causes endoplasmic reticulum and cytosolic stress, certain types of progeria, such as HGPS, exhibit nuclear proteotoxicity (Kubben & Misteli, 2017).

There are at least eleven phenotypically distinct single gene disorders, including both autosomal recessive and autosomal dominant disorders, due to genomic alterations in *LMNA*. To date, most progeroid‐associated LMNA mutations have been reported to be found primarily in the C‐terminal globular domain. A few scattered mutations in the rod‐1 and rod‐2 domains also result in a similar phenotype. The C‐terminal mutations of LMNA, such as C‐terminally truncated LMNA and G608S, do not lead to mislocalization of the protein (Kubben & Misteli, 2017). Instead, binding of these mutant LMNA proteins to the envelope results in irregularly shaped nuclei (Kubben & Misteli, 2017). Farnesylation of the C‐terminal residues of mutant LMNA may contribute to this process. Mutations such as A57P, L140R, etc. in the rod‐1 domain preclude dimerization of LMNA monomers, resulting in diffuse nucleoplasmic localization of LMNA (Casasola et al., 2016). However, some of the mutations such as S143P, E161K, etc. in the rod‐1 domain generate aggregation‐prone LMNA structures (West et al., 2016). On the other hand, the functions of the second rod domain of LMNA are unclear, and the effects of mutations in this region in respect to laminopathic disorders are not well characterized, although such mutations, such as D300G (Kane et al., 2013), have more severe effects on progeria.

Our data show that an E262K mutation in the rod‐2 domain collapses the helical structure in the mutation region. Because the conserved E262 residue is involved in a large number of inter‐residue interactions, this residue could be considered as an important node in the protein structure network of wild‐type LMNA. The E262K mutation in LMNA^E262K^ is destabilizing, possibly due to repulsive interactions of K262 with the similarly charged neighboring lysine residues (K260, K261, and K265). The repulsive and steric effects of the E262K mutation lead to a loss of interaction of K262 with the neighboring residues, allowing the region to undergo a transition from helix to disorder. Interestingly, the unfolded region reorganizes temporally such that several of the hydrophobic residues near the mutation are proximal to each other, forming two contiguous hydrophobic patches. The energetically unfavorable solvent‐exposed hydrophobic patches near the mutation of LMNA^E262K^ possibly undergo hydrophobic patch collapse and form aggregates. However, in wild‐type LMNA, these hydrophobic residues are dispersedly distributed by intermittent charged and polar residues. Given the energetic constraints, E262 of the wild‐type LMNA can only be replaced by an aspartic acid to maintain the essential salt bridges with K260, K261, and K265. Global level analysis revealed that substitution of this residue with another amino acid would essentially destabilize this region.

Disordered regions in multiple proteins, such as in TDP‐43, amyloid beta peptides, etc., have been reported to cause aggregation of the respective proteins (Uemura et al., 2018). While hydrophobic residues play a crucial role in this process (Fink, 1998), charged residues can also trigger aggregation through electrostatic interactions in some proteins, such as in FUS (Shelkovnikova et al., 2014). Aggregation of LMNA^E262K^ follows the former model of disorder and hydrophobicity for aggregation. This model of aggregation can be extrapolated to other LMNA mutants. For example, disruption of the helix by introduction of the helix‐breaking proline and glycine residues in certain mutations, such as in A57P, R60G, S143P, and D300G, could potentially cause destabilization and aggregation of LMNA in a manner similar to E262K. In contrast, mutations in the globular beta‐sheets or in the C‐terminal ‘SHG‐rich’ region have not been shown to cause aggregation. Although the liquid–liquid phase separation of LMNA^E262K^ in the nucleoplasm is evident, further studies on its amyloidogenic properties and nucleation steps could shed light on the generalized aggregation mechanisms of the rod domain‐associated mutations of LMNA.

Mutations in the rod domains of LMNA are known to induce aggregation properties of the protein. However, different mutations transform LMNA into different types of aggregation‐prone entities. For example, mutation of a basic or acidic amino acid to uncharged amino acids, such as D192G, H222P, R249W, and D446V, forms mild and smaller aggregates of the mutant LMNA. In contrast, mutation of residues to charged amino acids, such as L85R, E161K, E262K, and R386K, results in large nucleoplasmic aggregates of mutant LMNA. Moreover, some aggregate‐forming LMNA mutants remain in the nuclear lamina, whereas other aggregation‐prone LMNA mutants are completely mislocalized to the nucleoplasm. Interestingly, the aggregates of the different LMNA mutants are morphologically different. While some of the mutants form smaller, punctate aggregates, others form filamentous and globular aggregates. It is likely that specific mutations in LMNA trigger aggregation of the protein by different mechanisms. Although not much is known about the nucleation process of LMNA mutants, the aggregation of the phosphorylation‐deficient mutant (S143P) and the charged‐to‐nonpolar mutants demonstrate the importance of specific charged residues in maintaining the stability of LMNA. Loss of these charged residues could lead to a local change in hydrophobicity, resulting in aggregation of LMNA mutants in the aqueous nucleoplasm. Long‐distance electrostatic interactions may also play an enhancing role in the aggregation of the mutant LMNA proteins. Our study shows that the formation of disorder region and hydrophobic patches near the E262K mutation causes phase separation and aggregation of LMNA^E262K^. Although not deciphered, phase‐separated aggregates of LMNA^L85R^ and LMNA^R386K^ may form in the nucleoplasm by a mechanism similar to that of LMNA^E262K^. However, the formation of intermediate filaments of LMNA^E161K^ and nuclear speckles of LMNA^H222P^ could occur by different mechanisms. Nevertheless, aggregate‐forming LMNA mutants, with the exception of LMNA^E262K^, are involved in the development of dilated cardiomyopathy and Emery–Dreifuss muscular dystrophy 2. A previous study and we show that LMNA^E262K^ causes atypical progeria. The aggregation characteristics and clinical manifestations of LMNA mutant aggregates are summarized in Table 1.

**TABLE 1:** Aggregation‐prone mutants of LMNA and their clinical manifestations

Sl. No.	Mutation in LMNA	Expression pattern of mutant LMNA	Disease	Clinical phenotypes	References
1	p.(L85R)	Phase‐separated nucleoplasmic aggregates	Dilated cardiomyopathy (MIM# 115200)	Sinus bradycardia, atrioventricular conduction block, atrial arrhythmias	Boudreau et al. (2012) and Fatkin et al. (1999)
2	p.(S143P)	Nucleoplasmic localization and intranuclear aggregates	Dilated cardiomyopathy (MIM# 115200)	Progressive atrioventricular conduction defect, ventricular systolic dysfunction and dilatation, end‐stage heart failure, sudden death	Karkkainen et al. (2004) and Piekarowicz et al. (2017)
3	p.(E161K)	Intermediate filament disorganisation	Dilated cardiomyopathy (MIM# 115200)	Early atrial fibrillation, sinoatrial block, fibrosis of ventricle, congestive heart failure	Piekarowicz et al. (2017) and Sebillon et al. (2003)
4	p.(D192G)	Small number of large nucleoplasmic aggregate	Dilated cardiomyopathy (MIM# 115200)	Non‐specific myocyte damage, interstitial fibrosis	Boudreau et al. (2012) and Sylvius et al. (2005)
5	p.(H222P)	Speckle‐like aggregate in nuclear envelope	Emery‐Dreifuss muscular dystrophy 2 (MIM# 181350)	Muscle wasting and rigidity, Scapular winging, weak neck muscles, striated skeletal muscle defects	Bonne et al. (2000) and Piekarowicz et al. (2017)
6	p.(R249W)	Small puncta‐like scattered aggregates in nucleoplasm	Emery‐Dreifuss muscular dystrophy 2, (MIM# 181350)	Wasting/weakness of biceps, triceps, brachii	Scharner et al. (2011) and Steele‐Stallard et al. (2018)
7	p.(E262K)	Phase‐separated nucleoplasmic aggregates	Atypical progeria	Short stature, shallow orbits, narrow nasal bridge with broad nasal tip, craniofacial disproportion with micro‐retrognathia and dental crowding, dark fingernails with longitudinal ridges	This study
8	p.(R386K)	Aggregate‐like intra‐nuclear foci	Emery‐Dreifuss muscular dystrophy 2, (MIM# 181350)	Dilated cardiomyopathy and striated skeletal muscle defects and Dunnigan‐type partial lipodystrophy	Bonne et al. (2000) and Boudreau et al. (2012)
9	p.(D446V)	Mild nuclear aggregate	Emery‐Dreifuss muscular dystrophy 2, (MIM# 181350)	Proximal myopathy in elbow, ankle, neck	Piekarowicz et al. (2017) and Vytopil et al. (2003)

Posttranslational modifications of various residues of LMNA occur in response to cell stage or stress. LMNA is phosphorylated at several serine and threonine residues during mitotic division (Olsen et al., 2010), whereas several of the lysine residues are SUMOylated upon DNA damage and replication stress (Hendriks et al., 2015). SUMOylation of nuclear proteins, including LMNA, is redundantly mediated by the E2 SUMO ligase UBE2I. The E262K mutation in LMNA abolishes the consensus binding site of UBE2I (^259^YKKE^262^ to ^259^YKKK^262^). Surprisingly, this mutation completely destroys the binding potential of UBE2I to LMNA^E262K^, although there is another UBE2I binding site at ^200^MKEE^203^. Surprisingly, the ^200^MKEE^203^ region in rod‐1 is located exactly opposite to the ^259^YKKE^262^ region of rod‐2. Therefore, the UBE2I in the heterotrimeric complex of RanBP2/RanGAP1‐ SUMO/UBE2I could bind alternatively to both regions without being released from LMNA. Unfolding of the E262K mutation region potentially affects binding of the UBE2I complex to LMNA^E262K^ in a manner that also diminishes the binding potential of UBE2I to the ^200^MKEE^203^ region, resulting in possible loss of SUMOylation of lysine residues such as K201 and K260. The very low level of SUMOylation of LMNA^E262K^ may be due to the activity of an uncharacterized SUMO ligase of LMNA.

The function of SUMOylation of LMNA is elusive. It is possible that SUMOylation complements ubiquitination in terms of modulating the stability of LMNA. Our results indicate that loss of SUMOylation of LMNA^E262K^ prevents its degradation, which may also contribute to aggregation of the protein. A previous report suggests that SUMOylation of LMNA drives its degradation during nucleophagy (Li et al., 2019). Loss of SUMOylation at K201 also leads to the accumulation of LMNA^K201R^ in nucleoplasmic aggregates (Zhang & Sarge, 2008), suggesting that SUMOylation is not only required for the localization of LMNA in the nuclear envelope, but that loss of this modification also regulates the unusual accumulation of mutant LMNA under pathological conditions. SUMOylation of the LMNA tail is also impaired in partial lipodystrophy‐causing mutations (Simon et al., 2013). Consistent with these results, our finding highlights the need for proper SUMOylation of LMNA with respect to the localization specificity and degradation capacity of this protein. Additionally, the interplay of ubiquitination and SUMOylation may be an interesting aspect for understanding the spatiotemporal clearance of LMNA in normal and diseased conditions.

Mutation of LMNA as a cause of several premature aging disorders has long been known, although the molecular mechanisms underlining the link between mutant LMNA and progeria are not clear. Because many reports cite an imbalance in cellular proteostasis as a driver of aging in the organism, it was interesting to understand whether deregulated nuclear proteostasis due to mutant LMNA is a cause of early aging. Indeed, we observed a fundamental link between the laminopathy‐associated E262K mutation of LMNA and the induction of nuclear proteotoxicity. Aggregates of LMNA^E262K^ included essential chaperones, proteasomal proteins, etc., a phenomenon that not only disrupted protein folding but also thwarted the elimination of misfolded proteins and eventually generated further aggregates of nuclear proteins. Although nuclear protein aggregates were ubiquitinated, they were not efficiently degraded because of proteasome inactivation. Previously, aggregates of LMNA^Q432X^ were shown to sequester the transcription factor SREBP1 (Yang et al., 2013). In addition, a high‐throughput screening of interactors of different LMNA mutants revealed enrichment of several transcription factors such as zinc‐finger transcription factors (e.g., ZNF69, ZNF569, ZNF440, etc.) and CREB (Dittmer et al., 2014), indicating a definitive role of LMNA mutants in triggering transcriptional deregulation. While aberrant transcription in laminopathies would result in qualitative and quantitative alteration of the proteome, we note that nuclear proteotoxicity represents an additional stress that could act at the levels of protein folding and degradation.

The lethal effects of LMNA^E262K^ extend beyond proteotoxicity to impairment of DNA damage repair pathways. Like chaperones and proteasomal proteins, aggregates of LMNA^E262K^ sequester DNA damage repair proteins. Sequestration of DNA damage repair proteins by such aggregates would gradually reduce repair of spontaneously occurring DNA damage, leading to accumulation of extensive damaged DNA foci over time. Based on the observation that LMNA^E262K^ aggregates do not colocalize with DNA damage sites, we rule out the possibility that aggregated LMNA^E262K^ binds directly to DNA to cause the damage. Similar to LMNA^E262K^, condensed chromatin was previously observed in cells expressing LMNA^S143P^, although LMNA^S143P^ aggregates also did not bind to chromatin (West et al., 2016). Thus, the modulation of chromatin structure and impairment of the DNA repair process would be a passive effect due to the proteotoxicity of the aggregates of mutant LMNA.

In summary, we identified that E262K mutation in the rod‐2 domain of LMNA leads to early aging and that structural unfolding‐induced aggregation of the mutant LMNA causes severe proteotoxicity and failure of DNA damage repair, thus elucidating the mechanism of early aging due to an imbalance in nuclear proteostasis.

## 4 MATERIALS AND METHODS

Details of all methods and materials are provided in the Supporting Information.

## AUTHOR CONTRIBUTIONS

DKG: Conceptualization, Methodology, Resource acquisition, Investigation, Formal analysis, Validation, Data curation, Writing original draft. SP: Methodology, Investigation, Formal analysis, Writing original draft. JK: Methodology, Formal analysis. DY: Clinical evaluation. PR: Methodology. SN: Clinical evaluation. CGR: Methodology, Software, Validation, Writing original draft. AR: Resource acquisition, Formal analysis, Validation. KMG: Conceptualization, Resource acquisition, Clinical evaluation, Formal analysis, Validation, Project administration, Funding acquisition and overall supervision.

## CONFLICT OF INTEREST

KMG is founder and director of Suma Genomics Private Limited, interested in rare disease diagnosis. Other authors report no conflict of interest.

## Funding information

This work was supported by the DBT/Wellcome Trust India Alliance grant [Grant number: IA/CRC/20/1/600002] awarded to KMG. Shruti Pande is supported by the Nurturing Clinical Scientist fellowship (Grant number: HRD/Head NCS‐2019‐03) from Indian Council for Medical Research, New Delhi.

## PATIENT CONSENT STATEMENT

The patient and his parents provided written informed consent to the study.

## Supporting information


**Appendix S1.** Supplementary Material.

## Data Availability

All materials and data of this study are available from the corresponding authors upon request.
