# Supplementary material for: The E262K mutation in Lamin A links nuclear proteostasis imbalance to laminopathy‐associated premature aging
Source: Aging Cell. 2022 Oct 12;21(11):e13688. doi: 10.1111/acel.13688 (PMC9649601; doi:10.1111/acel.13688)
Supplement: Supplementary file 1 — Appendix S1. Supplementary Material. [file ACEL-21-e13688-s001.pdf]

Supporting information for

The E262K mutation in lamin A links nuclear proteostasis imbalance to laminopathy-associated premature aging

Debasish Kumar Ghosh[1,*,$], Shruti Pande[1,*], Jeevan Kumar[1], Dhanya Yesodharan[2], Sheela Nampoothiri[2], Periyasamy Radhakrishnan[3], Chilakala Gangi Reddy[4], Akash Ranjan[4], Katta M. Girisha[1,3,$]

[1]Department of Medical Genetics
Kasturba Medical College, Manipal
Manipal Academy of Higher Education
Karnataka 576104, India

[2]Department of Pediatric Genetics
Amrita Institute of Medical Sciences & Research Centre
Cochin 682041, Kerala, India

[3]Suma Genomics Private Limited and Manipal Center for Biotherapeutics Research
Manipal Academy of Higher Education
Karnataka 576104, India

[4]Computational and Functional Genomics Group
Centre for DNA Fingerprinting and Diagnostics
Hyderabad 500039, Telangana, India

* Equal contribution

[$] To whom correspondence shall be addressed

Email: debasish.kumar@manipal.edu, girish.katta@manipal.edu

Table of Contents

Page number

(1) Materials and methods 3-10
(2) Table S1 11
(3) Table S2 12
(4) Figure S1 13
(5) Figure S2 14
(6) Figure S3 15
(7) Figure S4 16
(8) Table S3 17

Materials and methods

Family ascertainment

The proband is from a family from southern part of India. We obtained informed medical photography consent and consent for skin biopsy from the parents of the proband for all the clinical tests and related studies. The research was reviewed, with a written approval from the duly commissioned Katurba Medical College and Kasturba Hospital institutional ethics committee (Ethical clearance number: 383/2020).

Whole exome sequencing and variant filtering

Genomic DNA was extracted (Qiagen, 51104) from the blood cells of proband and parents. Singleton exome sequencing was performed for the proband. The exonic and flanking genomic regions were captured using TWIST Humancore capture kit (Twist Biosciences). Exome sequencing had an average coverage of 100 to 130X, 95% of bases covered at a minimum of 20X with 97% sensitivity (1). Raw data were retrieved in FASTQ format and aligned to GRCh38 assembly using Burrows-Wheeler Aligner (v0.7.15) and our in-house pipeline based on 'Genome Analysis Toolkit Best Practices'. These data were annotated by 'Annotate Variation (ANNOVAR)' (2) and our in-house scripts. The filtered variants were analyzed using variant prioritization and filtering strategy (3) as outlined in supporting information **Table S3**.

Cloning

Total RNA was isolated (ThermoFischer Scientific [TFS], 12183018A) from HEK293T cells, followed by generation of cDNAs from mRNAs of LMNA and UBE2I by reverse transcription (TFS, 4368814) using oligo-dT primer (Eurofins Genomics [EG]). The list of clones and plasmids used in this study can be found in **Table 1**. Open reading frames of LMNA and UBE2I were amplified by PCR (New England Biolabs Inc [NEB], M0531S) using the primer sets (EG) listed in **Table 2**. The overall gene cloning process was similar to our previous studies (4, 5). The PCR products and expression vector were digested with restriction enzymes (NEB), followed by ligation (TFS, K1422) of the restriction-digested PCR products with the vector. The ligated products were transformed into the ultracompetent DH5α strain of *Escherichia coli*, followed by selection of positive colonies (clones) by colony PCR (Takara Bio, RR350A).

Mutagenesis:

The point mutation (c.784G>A, corresponding to E262K) in the LMNA ORF was introduced using specific primer sets (**Table 2**) in an overlapping PCR process. The ORFs, corresponding to the rod-2 domain (226-387 amino acid region) of LMNA and LMNAE262K, were cloned using specific primer sets (**Table 2**) with the cloning procedure mentioned above. The sequences of all clones were verified at the Research Support Service Group of CDFD.

Table 1. Clones and plasmids

Clone/Plasmid	Source	Identifier
Bacterial expression		
LMNA_pET21b	Present study	
LMNA^{E262K}_pET21b	Present study	
LMNA-rod2_pET21b	Present study	
LMNA^{E262K}-rod2_pET21b	Present study	
UBE2I_pET21b	Present study	

FLAG_UBE2I_pcDNA3.1	Present study	
pcDNA3.1	ThermoFischer Scientific	V790-20
pET21b	Novagen	69741-3

Table 2. Oligonucleotides

No.	Clone	Primer	Sequence
	**Cloning primers**		
1	LMNA_pET21b	Forward	GGAATTCCATATGCATCATCATCATCATCATGAGACCCCGT CCCAGCGGCGCGCCACC
		Reverse	CCGCTCGAGTTACATGATGCTGCAGTTCTGGGGGCTCTGG GTTC
2.	LMNA^{E262K}_pET21b	Forward-1	GGAATTCCATATGCATCATCATCATCATCATGAGACCCCGT CCCAGCGGCGCGCCACC
		Reverse-1	AATAAGTCTTCTCCAGCTTCTTCTTATACTGCTCCA
		Forward-2	TGGAGCAGTATAAGAAGAAGCTGGAGAAGACTTATT
		Reverse-2	CCGCTCGAGTTACATGATGCTGCAGTTCTGGGGGCTCTGG GTTC
3.	LMNA-rod2_PET21b	Forward	GGAATTCCATATGGTGGAGATTGACAATGGGAAGCAGCG TGAG
		Reverse	CCGCTCGAGTAGCCTCTCCTCCTCGCCCTCCAAGAGCTTGC G
4.	LMNA^{E262K}-rod2_PET21b	Forward	GGAATTCCATATGGTGGAGATTGACAATGGGAAGCAGCG TGAG
		Reverse	CCGCTCGAGTAGCCTCTCCTCCTCGCCCTCCAAGAGCTTGC G
5.	UBE2I_pET21b	Forward	GGAATTCCATATGTCGGGGATCGCCCTCAGCAGACTCGCC CAGG
		Reverse	CCGCTCGAGTGAGGGCGCAAACTTCTTGGCTTGTGCTCG
6.	FLAG_UBE2I_pcDNA3.1	Forward	GGAATTCCATATGGACTACAAAGACGATGACGACAAGTC GGGGATCGCCCTCAGCAGACTCGCCCAGG
		Reverse	CCGCTCGAGTTATGAGGGCGCAAACTTCTTGGCTTGTGCT CG
	**qPCR primers**		
6	LMNA	Forward	AAGCAGCGTGAGTTTGAGA
		Reverse	CCTCTCAGCAGACTGCCTGG
7	LMNA-ORF	Forward	ATGCATCATCATCATCATCATGAGACCCCGT
		Reverse	TTACATGATGCTGCAGTTCTGGGGGCTCTGG

Production and purification of recombinant proteins

Recombinant LMNA, LMNA^{E262K}, LMNA-rod2, LMNA^{E262K}-rod2, and UBE2I proteins were produced using T7 expression in a procedure we described in previous studies (6, 7). Bacterial expression clones were transformed into the BL21DE3 strain of Escherichia coli followed by induction of protein expression by 1mM isopropyl-β-D-1-thiogalactopyranoside (IPTG, Sigma Aldrich [SA], I6758) in culture medium (Luria-Bertani broth [Himedia, M1245]/ampicillin [SA, A9393]) for 12 hours at 37°C, 180 rpm. Bacterial cells were harvested (5000 g, 37°C, 15 min) and resuspended in pre-chilled lysis buffer (50 mM Tris-HCl, pH 8.0, 300 mM NaCl, 10 mM imidazole, 1 mM PMSF (SA, P7626). Precleared (12000 g, 4°C, 40min.) cell lysate was passed through a column containing Ni^{+2}-NTA agarose beads (Qiagen [QA], 30250), followed by repeated washing of Ni^{+2}-NTA agarose beads bound to recombinant

protein with wash buffer (50 mM Tris-HCl, pH 8.0, 300 mM NaCl, 40 mM imidazole) and elution of the recombinant proteins in elution buffer (50 mM Tris-HCl, pH 8.0, 300 mM NaCl, 300 mM imidazole). Proteins were dialyzed in different dialysis buffer depending upon the downstream experimental requirements. The quality of purified proteins was checked using SDS-PAGE, and > 98% pure proteins were used for the experiments.

Circular dichroism spectroscopy

Analysis of the secondary structure of LMNA and LMNAE262K was performed by circular dichroism spectroscopy (CD) using the JASCO-810 spectropolarimeter equipped with a Peltier temperature controller. Proteins were kept in analysis buffer (20mM NaH_2PO_4, pH 7.4, 50mM NaF). Changes in ellipticity values ($\Delta\varepsilon$) of 1 µM proteins were recorded in the far UV range (190-260 nm) of light. The following instrument parameters were used: Light path length: 0.1 cm, wavelength scan speed: 100 nm/min, response time: 1 sec, data pitch: 1 nm, band width: 2 nm. In the thermal unfolding experiments, the proteins were slowly heated from 20°C to 90°C with a gradient of 1°C/min, and the $\Delta\varepsilon$ values were recorded at a light wavelength of 222 nm. The secondary structure components were analyzed using the CD spectra. From the CD spectra, the secondary structure components of the different proteins were analyzed in the DichroWeb platform using the CDSSTR algorithm (8). The melting temperature of the proteins was analyzed using the Denaturation Analysis program of the Spectra Manager.

Atomic force microscopy

The height distribution of protein particles was measured by atomic force microscopy (AFM). 2 µl of a protein solution (in deionized filtered water) was drop-casted over an atomically flat mica sheet, followed by air drying of the protein solution in a dust-free chamber at 25°C. The protein was mildly washed with deionized filtered water and then dried with a gentle stream of nitrogen gas. *Ex situ* AFM images were acquired using a Vecco di INNOV (Bruker) instrument equipped with a large area scanner. Instrument parameters included: - resonance frequency: 225-300 KHz, free oscillation amplitude: 5 V, set amplitude: 2.9 V, and scan speed rate: 0.6-0.8 Hz. Image preparation and height distribution analysis of particles were performed using Gwyddion (9) and WSxM 4.0 Beta 7.0 (10) software, respectively.

Dynamic light scattering

The hydrodynamic diameter of protein particles (in 20mM Tris-Cl, pH 7.4, 50mM NaCl) at different concentrations was measured using Malvern particle size analyzer (Zetasizer Nano ZS90, Malvern Panalytical). Particle diameter analysis was performed using 21 CFR Part11 software.

Isothermal titration calorimetry

Binding assays of LMNA and $LMNA^{E262K}$ with UBE2I were performed by isothermal titration calorimetry in a Microcal iTC 2000 (GE Healthcare) instrument with Microcal-Thermovac temperature control system. The general process of ITC was similar to our earlier studies (11, 12). All proteins were in binding buffer (10mM Tris-Cl, pH 8.0, 20mM NaCl) at 25°C. The cell contained 1 µM LMNA or $LMNA^{E262K}$, while the syringe contained 20 µM UBE2I. The following instrument parameters were used in the binding experiments - number of binding events: 20 (first injection volume of 0.4 µl for 0.8 sec; remaining nineteen injections of 2.0 µl for 4.0 sec), filter period: 5, syringe rotation speed: 200 rpm, instrument reference power: 10 µcal/sec. Data acquisition and analysis were performed in Origin graphics software, and dissociation constants (K_d) were calculated considering the 1:1 binding stoichiometry of binding.

Cell culture

Fibroblast cells were obtained from the thigh and abdomen of the proband with informed consent and institutional ethical committee approval. Fibroblasts from an age-matched, control individual were obtained from our in-house cell collection. For all the experiments, fibroblasts were chosen because lamin A is sufficiently expressed in fibroblasts. Monitoring the level and expression of LMNA and LMNAE262K and their interplay with other nuclear proteostasis and DNA repair proteins was easy to follow in fibroblasts. Moreover, fibroblasts were easily obtainable from control individual and proband. Therefore, we followed the recommended use of fibroblasts for progeroid-related studies and understanding of cellular proteostasis. We chose fibroblasts of two regions, thigh, and abdomen, of the proband in order to understand if the aggregation phenotype of LMNAE262K is consistent in different regions of body of proband.

Cells were cultured in advanced DMEM medium (TFS, 12491023) supplemented with 10% fetal bovine serum (TFS, 26140095), 2 mM L-glutamine (TFS, 25030164), and 1x antibiotic-antifungal solution (TFS, 11548876). Cell cultures were maintained optimally in a sterile, humidified incubator at 37°C and 5% CO_2. For specific experiments, control and proband fibroblasts were treated with 100nM cycloheximide (SA, 01810).

Transfection: 60% confluent monolayer fibroblasts were serum starved for 2 h before the transfection. 16 µg of DNA was added to 100 µl of Opti-MEM (TFS, 31985062) with 6 µl of Lipofectamine2000 (TFS, 11668019) (optimized for a 100 mm culture plate; DNA and Lipofectamine2000 were scaled up or down depending upon the surface area of culture dish). The transfection complex was kept at room temperature for 20 minutes, followed by addition of 3 µl chitosan (SA, 448869) aqueous acetic acid solution (5 mg/ml) to the transfection complex solution. The transfection complex was further diluted in 2 ml of Opti-MEM containing 5 µl DMSO (SA, D2650). The final solution was poured over the PBS washed cells, and kept at 37°C, 5% CO_2 for 6 h. Following the transfection period, cells were washed with PBS and cultured with optimal growth medium. An overall 70-85% transfection efficiency was achieved.

Immunoprecipitation and immunoblotting

Immunoprecipitation: LMNA and $LMNA^{E262K}$ were immunoprecipitated from proband and control fibroblasts using anti-LMNA antibody (see **Table 3**). Cells were lysed in prechilled RIPA buffer (TFS, 89900) with 1% SDS for 15 min at 4°C, followed by removal of debris from the lysate (14000 g, 4°C, 20 min). 2 mg of total protein in the lysate was used for denaturing immunoprecipitation using the Crosslink Magnetic IP/co-IP method (TFS, 88805; Pierce Crosslink Magnetic IP/co-IP Kit) following manufacturer's protocol. Briefly, 10 µg of anti-LMNA antibody was bound to protein A/G magnetic beads in 100 µl reaction volume for 15 minutes at 25°C. Antibody was crosslinked to the protein A/G magnetic beads in coupling buffer containing 20 µM DSS (crosslinker), followed by collection of antibody bound protein A/G magnetic beads by centrifugation (5000 g, 5 min., 4°C). 2 mg total protein in 500 µl cell lysate was incubated with the antibody-bound protein A/G magnetic beads on a rotative wheel at 4°C for 12 h, followed by precipitation of the beads by centrifugation (5000 g, 5 min., 4°C). Beads were successively washed by wash buffer and antibody-bound LMNA was eluted in elution buffer. The elution buffer was neutralized by neutralization buffer.

Immunoblotting: 60 µg of protein in the cell lysate was mixed with 6x Laemmli buffer (TFS, ALF-J61337-AC) and the suspension was heated to 95°C for 10 min. Proteins were separated in 8% SDS-PAGE and then transferred to a polyvinylidene fluoride (PVDF) membrane (Amersham Hybond P 0.45; GE Healthcare Life Sciences, 10600023). The membrane was then blocked with 5% skim milk solution (in

Tris buffer saline [TBS], TFS, 28358), followed by application of the primary and secondary antibodies (see **Table 3**) and intermittent washing with TBST buffer (TBS with 0.1% Tween20). SuperSignal west femto maximum sensitivity substrate was used to generate the chemiluminescent signals, which were detected using the ChemiDoc XRS+ Imaging System (Bio-Rad). Densitometric quantification of protein bands in immunoblots was performed using ImageJ2 software.

Table 3. Antibodies

Primary antibodies			
**Target protein**	**Species**	**Supplier**	**Catalogue number**
Histone H3	Rabbit	Abcam	ab18521
HSPA1A	Mouse	Abcam	Ab2787
LMNA	Rabbit	Sigma-Aldrich	L1293
MRE11	Mouse	Abcam	ab214
$p16^{INK4a}$	Rabbit	Thermo Fischer Scientific	MA5-32133
$p21^{waf1/cip1}$	Rabbit	Cell Signaling Technology	2947
pS139-H2A.X	Mouse	Merck Millipore	05-636
PSMD8	Mouse	Santa Cruz Biotechnologies	sc-514053
SUMO2	Rat	Sigma-Aldrich	SAB4200190
UBE2I	Mouse	Sigma-Aldrich	SAB5300006
Ubiquitin	Mouse	Cell Signalling Technology	3936
XRCC5	Goat	Sigma-Aldrich	SAB2500579
Secondary antibodies (Immunoblotting)			
anti-rabbit IgG (whole molecule) peroxidase antibody	Goat	Sigma-Aldrich	A0545
anti-mouse IgG (whole molecule) peroxidase antibody	Rabbit	Sigma-Aldrich	A9044
anti-rat IgG (whole molecule) peroxidase antibody	Rabbit	Sigma-Aldrich	A5795
Secondary antibodies (Immunocytochemistry)			
anti-Goat IgG (H+L)-Alexa Fluor Plus 647	Donkey	ThermoFischer Scientific	A-21447
anti-Mouse IgG (H+L)-Alexa Fluor Plus 555	Goat	ThermoFischer Scientific	A32727
anti-Rabbit IgG (H+L)-Alexa Fluor Plus 488	Goat	ThermoFischer Scientific	A32731
anti-Rat IgG (H+L)-Alexa Fluor Plus 647	Goat	ThermoFischer Scientific	A48265

Immunocytochemistry and fluorescence microscopy

Adherent cells grown on glass coverslips (Blue Star) were washed with phosphate buffer saline (PBS, pH 7.4; TFS, 10010023), followed by fixation of the cells with 4% paraformaldehyde (SA, 158127) solution (in PBS) for 15 min at 25°C. Cells were permeabilized with 0.2% Triton X-100 (SA, T8787) solution (in PBS) for 10 min at 25°C and then blocked with 1% bovine serum albumin (SA, B6917)

solution (in PBS) for 30 min at 25°C. Cells were intermittently washed with PBS. Cells were treated sequentially with primary antibodies (solubilized in 1% BSA-PBS) for 2 h at 25°C) and secondary antibodies (solubilized in 1% BSA-PBS) for 2 h at 25°C) (see **Table 3**) and washed with PBS, blocked with 1% BSA solution (in PBS) for 30 min at 25°C in between. Finally, the cells were mounted on glass slides with prolong antifade gold with or without DAPI (TFS, P36931; P10144).

Proteostat (Enzo lifesciences, ENZ-51035-0025) staining was performed on adherent-fixed cells according to the manufacturer's protocol.

Fluorescence images were acquired using LSM700 confocal laser scanning microscope (Carl Zeiss) with a 63x plan apochromat/1.4 NA oil/DIC M27 objective. Image processing was performed using Zen-Lite 2010 software (Carl Zeiss).

Colocalization of proteins was analyzed using the Coloc 2 plugin in FIJI. Colocalization of pixel intensities of proteins at different region of interests (ROI), such as in nuclear envelope/lamina, was measured by correlation threshold of Coloc 2. The Pearson correlation coefficient measured the colocalization of proteins in different samples. At least 25 cells were analyzed to measure the mean correlation coefficient of colocalization.

The pS139-H2A.x-positive damaged DNA foci in cells were manually counted.

Computational studies

Protein structure: the *ab initio* model structure of LMNA was generated in ROSETTA. The E262K mutation in the model structure of LMNA was generated using the Mutagenesis wizard of Pymol with the best oriented rotamer of lysine at position 262. The model structures of LMNA and LMNA^{E262K} were checked and validated in MolProbity (13), and the Ramachandran plots of both structures were checked. The LMNA-rod2 and LMNA^{E262K}-rod2 structures were extracted from LMNA and LMNA^{E262K} structures respectively.

Sequence conservation analysis: the Gremlin program (14) was used to understand amino acid conservation and coevolution. The conservation score measured the linkage matrix of amino acids of wild-type LMNA of different organisms. The residues with a bit score of at least 1 are considered conserved.

Molecular dynamics simulation

Molecular dynamics simulations of LMNA-rod2 and LMNA^{E262K}-rod2 were performed using GROMACS/2018.8 (15). The topology and GROMACS-readable coordinate files of the protein were generated using the OPLS-AA /L forcefield (16) and the TIP4P (17) water model. The system was then constructed by defining a triclinic unit cell, since the protein domain is rod-shaped. The protein was then placed in the center of the unit cell with a minimum distance of 1.0 nm between the edges of the protein and the unit cell. The unit cell was solvated with the TIP4P water model, along with a salt concentration of 0.15 M. The charge of the system was neutralized with sodium (Na) ions.

The system was relaxed, and steric clashes were resolved by energy minimization using the steepest-descent algorithm. The system was then equilibrated in an isothermal-isochoric ensemble (NVT ensemble with constant particle number, volume, and temperature) at a temperature of 300 K using the V-rescale temperature coupling method (18). The system was again equilibrated under isobaric-isothermal conditions (NPT ensemble with constant particle number, pressure, and temperature) at an atmospheric pressure of 1.0 bar using the isotropic Parrinello-Rahman pressure coupling scheme (19). Both equilibration steps were performed using the Leap-Frog integrator with a

time step of 2 fs for 1 ns, LINCS constraints for bound parameters (20), Verlet cut-off for unbound parameters (21), periodic boundary conditions, and particle mesh Ewald for long-range electrostatics (22). Unconstrained production simulation of the equilibrated system was performed for 50 ns with a time step of 2 fs, with coordinates, velocities, and energy values written out every 10 ps.

The simulated system was post-processed using 'gmx trjconv' for correction of diffused protein coordinates and for centering in the unit cell. Then, the processed trajectories were analyzed with 'gmx rms', 'gmx rmsf', 'gmx hbond' and 'gmx gyrate' to understand the changes in root mean square deviation (RMSD), root mean square fluctuation (RMSF), hydrogen bonding and gyration radius, respectively. The changes in secondary structures were analyzed with 'gmx do_dssp' using the standalone Dictionary Secondary Structure of Protein (DSSP) program (23). Contact maps of the residues were generated using 'gmx mdmat'. Postscript files were generated from the output matrix files of 'gmx do_dssp' and 'gmx mdmat' using 'gmx xpm2ps'. To visualize changes in the tertiary structure, snapshots were extracted every 500 ps from the trajectory files using 'gmx trjconv'.

Statistical analysis

Quantitative data are presented as mean ± standard deviation. Statistical significance of difference between mean values of groups are analyzed by two-tailed, homoscedastic student's t-test. $P<0.05$ represented statistically significant difference.

Graphics

The graphics were made in Adobe Illustrator.

Reference

1. Girisha KM, von Elsner L, Neethukrishna K, Muranjan M, Shukla A, Bhavani GS, Nishimura G, Kutsche K, Mortier G (2019) The homozygous variant c.797G>A/p.(Cys266Tyr) in PISD is associated with a Spondyloepimetaphyseal dysplasia with large epiphyses and disturbed mitochondrial function. *Hum Mutat* 40: 299-309
2. Wang K, Li M, Hakonarson H (2010) ANNOVAR: functional annotation of genetic variants from high-throughput sequencing data. *Nucleic Acids Res* 38: e164
3. Pande S, Radhakrishnan P, Shetty NM, Shukla A, Girisha KM (2021) Hedgehog acyl-transferase-related multiple congenital anomalies: Report of an additional family and delineation of the syndrome. *Am J Med Genet A* 185: 2756-2765
4. Ghosh DK, Ranjan A (2021) HYPK coordinates degradation of polyneddylated proteins by autophagy. *Autophagy*: 1-22
5. Ghosh DK, Roy A, Ranjan A (2018) Aggregation-prone Regions in HYPK Help It to Form Sequestration Complex for Toxic Protein Aggregates. *J Mol Biol* 430: 963-986
6. Ghosh DK, Kumar A, Ranjan A (2018) Metastable states of HYPK-UBA domain's seeds drive the dynamics of its own aggregation. *Biochim Biophys Acta Gen Subj* 1862: 2846-2861
7. Ghosh DK, Roy A, Ranjan A (2018) The ATPase VCP/p97 functions as a disaggregase against toxic Huntingtin-exon1 aggregates. *FEBS Lett* 592: 2680-2692
8. Compton LA, Johnson WC, Jr. (1986) Analysis of protein circular dichroism spectra for secondary structure using a simple matrix multiplication. *Anal Biochem* 155: 155-67
9. Nečas D, KlapetekP (2012) Gwyddion: an open-source software for SPM data analysis, *Cent. Eur. J. Phys.* 10(1) 181-188
10. Horcas I, Fernandez R, Gomez-Rodriguez JM, Colchero J, Gomez-Herrero J, Baro AM (2007) WSXM: a software for scanning probe microscopy and a tool for nanotechnology. *Rev Sci Instrum* 78: 013705
11. Kumar A, Ghosh DK, Ranjan A (2020) Mefloquine binding to human acyl-CoA binding protein leads to redox stress-mediated apoptotic death of human neuroblastoma cells. *Neurotoxicology* 77: 169-180

12. Kumar A, Ghosh DK, Ali J, Ranjan A (2019) Characterization of Lipid Binding Properties of Plasmodium falciparum Acyl-Coenzyme A Binding Proteins and Their Competitive Inhibition by Mefloquine. *ACS Chem Biol* 14: 901-915
13. Chen VB, Arendall WB, 3rd, Headd JJ, Keedy DA, Immormino RM, Kapral GJ, Murray LW, Richardson JS, Richardson DC (2010) MolProbity: all-atom structure validation for macromolecular crystallography. *Acta Crystallogr D Biol Crystallogr* 66: 12-21
14. Ovchinnikov S, Kamisetty H, Baker D (2014) Robust and accurate prediction of residue-residue interactions across protein interfaces using evolutionary information. *Elife* 3: e02030
15. Abraham MJ, Murtola T, Schulz R, Páll S, Smith JC, Hess B, & Lindahl E (2015) GROMACS: High performance molecular simulations through multi-level parallelism from laptops to supercomputers. *SoftwareX*, 1–2, 19–25
16. Kaminski GA, Friesner RA, Tirado-Rives J, Jorgensen WL (2001) Evaluation and Reparametrization of the OPLS-AA Force Field for Proteins via Comparison with Accurate Quantum Chemical Calculations on Peptides. *The Journal of Physical Chemistry B*, 105(28), 6474–6487
17. Lawrence CP, Skinner JL (2003) Flexible TIP4P model for molecular dynamics simulation of liquid water. *Chemical Physics Letters*, 372(5), 842–847
18. Bussi G, Donadio D, Parrinello M (2007) Canonical sampling through velocity rescaling. *The Journal of Chemical Physics*, 126(1), 014101
19. Berendsen HJC, Postma JPM, van Gunsteren WF, DiNola A, Haak JR (1984) Molecular dynamics with coupling to an external bath. *The Journal of Chemical Physics*, 81(8), 3684–3690
20. Hess B, Bekker H, Berendsen HJC, Fraaije JGEM. (1997) LINCS: A linear constraint solver for molecular simulations. *Journal of Computational Chemistry*, 18(12), 1463–1472
21. Verlet L (1967) Computer "Experiments" on Classical Fluids. I. Thermodynamical Properties of Lennard-Jones Molecules. *Physical Review*, 159(1), 98–103
22. Darden T, York D, Pedersen L (1993) Particle mesh Ewald: An N·log(N) method for Ewald sums in large systems. *The Journal of Chemical Physics*, 98(12), 10089–10092
23. Kabsch W, Sander C (1983) Dictionary of protein secondary structure: Pattern recognition of hydrogen-bonded and geometrical features. *Biopolymers*, 22(12), 2577–2637

Table S1. List of rare exonic and splicing heterozygous variants observed in the proband with phenotypes indexed on OMIM. The variant in *LMNA* is highlighted.

Sl. No.	Chr:Start-Ref-Alt	Gene
1	1:10403337-C-G	*KIF1B*
2	1: 52420353- G- A	*LMNA*
3	1: 60628541- G- A	*HMCN1*
4	1: 32552055- GGC--	*FLVCR1*
5	1: 32552059- C- G	*RYR2*
6	2: 2552946- A--	*RANBP2*
7	2: 44153697- C- T	*RANBP2*
8	3:75614494- G- A	*DNAH1*
9	5: 80300475- -GGC	*ZSWIM6*
10	6: 32613992-- TCCTCCT	*HLA-DRB1*
11	6: 91963044-- TTATGGAAGTGTCTCTCCAGGAACCGCACCCGCTCCGTCCCA TTGAAGAAATGACACTCAGACTTAAGCAGCTCCAAGAAACG TGCTGTGGGGACACGAATGCTCCGGTCACACAGGCGGCCTC CTGAGAAGACACTGACAGCGACGCCGCCATCCGGGGCCCCC TGGGCCGGGTGCGGGCACTGGGA	*HLA-DRB1*
12	6: 135946936-- A	*HLA-DRB1*
13	7:110814651- G--	*LFNG*
14	7: 76578738- TGGAGCCCGAGTTTGAGACCCAGT- T	*AEBP1*
15	7: 78858141- C-T	*POR*
16	7:89802005- AAG- -	*CD36*
17	8: 50745669- T-C	*NRG1*
18	9: 17306064- TG--	*SECISBP2*
19	9: 41921255- CCCACGGGTGACTCCGGCGCCCCCCCCGTGCCGCCCACGGG TGACTCCGGGGCCCCCCCCGTGACC--	*CEL*
20	13: 110814651- C-T	*COL4A1*
21	15: 76578738- C-A	*ETFA*
22	15: 78858141 – C-T	*CHRNA5*
23	15: 89802005- AAGGTAATAAT--	*FANCI*
24	16: 50745669- C-A	*NOD2*
25	19: 41921255- C-A	*MYO9B*
26	22: 41921255- A-G	*ACO2*

Table S2. Variant summary of the *LMNA* gene in proband.

[LMNA] Variant Summary			
Known variant?	--		
Genomic coordinate	1:156104740		
Ref/Alt	G/A		
Zygosity	Heterozygous		
cDNA change	c.784G>A		
Protein change	p.(Glu262Lys)		
Variant effect	Misssense		
Gene details			
Gene name	*LMNA*		
Exon number	4		
Transcript ID	NM_170707.4		
Confidence scores for variant reads			
Read number	91 (42,49)	Quality	99
Frequency in the general population			
Variant frequency	gnomAD: Not present<br>In-house: Not present		
In-silico predictions			
CADD_phred	34	GERP++	5.58
REVEL	0.922	M-CAP	Possibly pathogenic
MutationTaster	Disease causing		
OMIM disease			
Hutchinson-Gilford progeria syndrome			

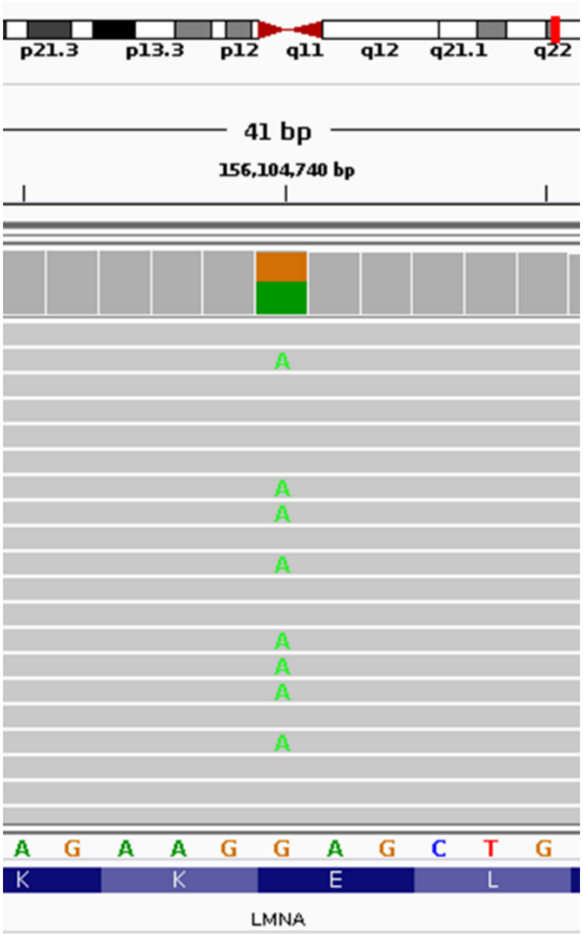

Figure S1. Integrated genomic viewer (IGV) snapshot showing the c.784G>A variant in heterozygous state in the proband.

GRCh37/hg19.1:156,104,740 Reference Allele G

Alt Allele	M-CAP	95% sensitivity
A	0.759	Possibly Pathogenic
C	0.378	Possibly Pathogenic

BayesDel addAF<br>*dbNSFP version 4.1*	addAF prediction<br>Damaging
BayesDel noAF<br>*dbNSFP version 4.1*	noAF prediction<br>Damaging
DANN<br>*version 2014*	Score<br>0.9994
DEOGEN2<br>*dbNSFP version 4.1*	prediction<br>Damaging
EIGEN<br>*dbNSFP version 4.1*	prediction<br>Pathogenic
EIGEN PC<br>*dbNSFP version 4.1*	prediction<br>Pathogenic
FATHMM<br>*dbNSFP version 4.1*	prediction<br>Damaging
FATHMM-MKL<br>*dbNSFP version 4.1*	coding prediction<br>Damaging
FATHMM-XF<br>*dbNSFP version 4.1*	coding prediction<br>Damaging
MVP<br>*dbNSFP version 4.1*	prediction<br>Pathogenic
MetaLR<br>*dbNSFP version 4.1*	prediction<br>Damaging
MetaSVM<br>*dbNSFP version 4.1*	prediction<br>Damaging
MutPred<br>*dbNSFP version 4.1*	prediction<br>Pathogenic
Mutation assessor<br>*dbNSFP version 4.1*	prediction<br>High
MutationTaster<br>*dbNSFP version 4.1*	Prediction<br>Disease causing
PROVEAN<br>*dbNSFP version 4.1*	prediction<br>Damaging
REVEL<br>*dbNSFP version 4.1*	prediction<br>Pathogenic

Figure S2. Predicted effect of the c.784G>A mutation in LMNA.

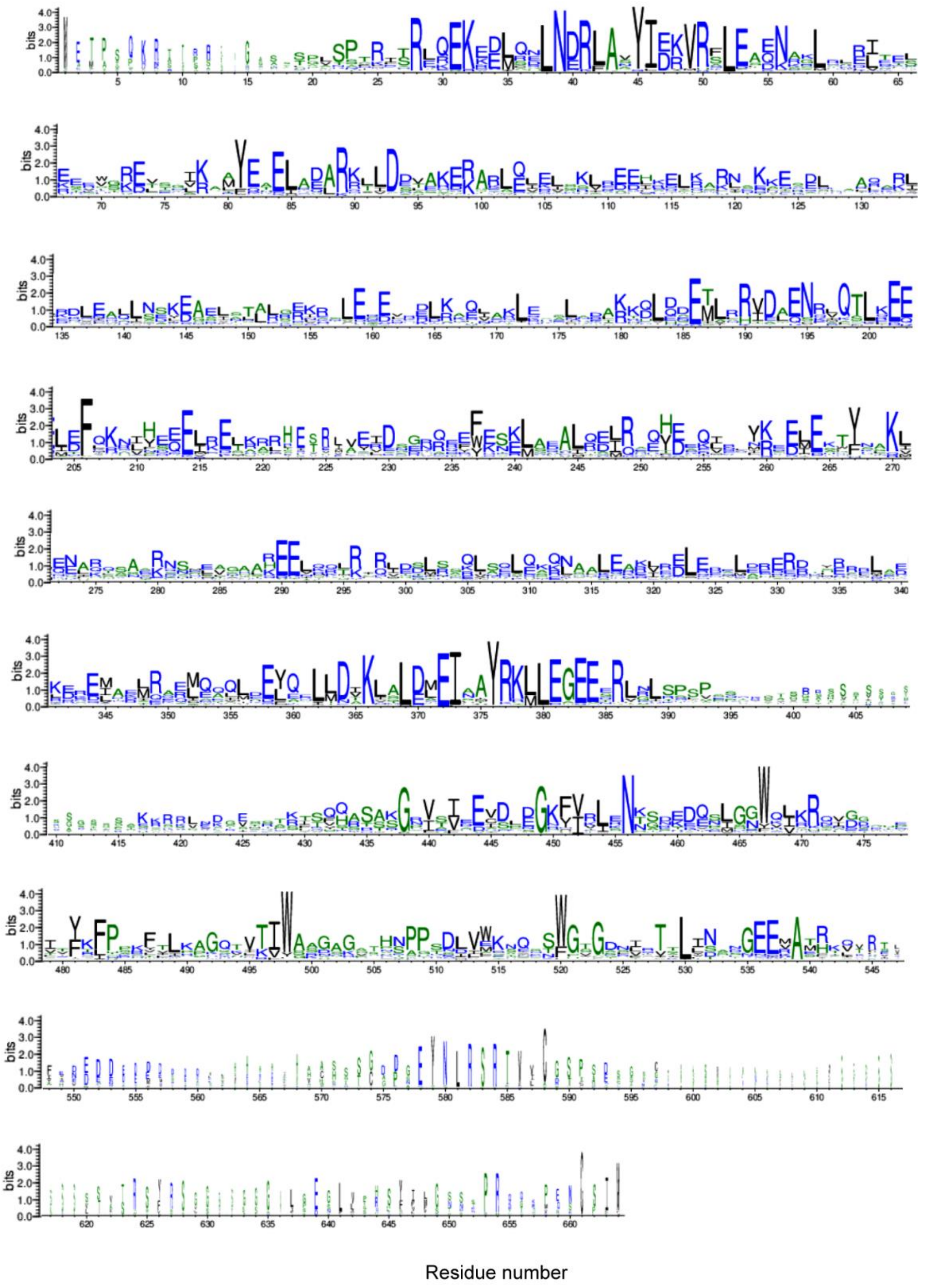

Figure S3. Sequence conservation plot of LMNA.

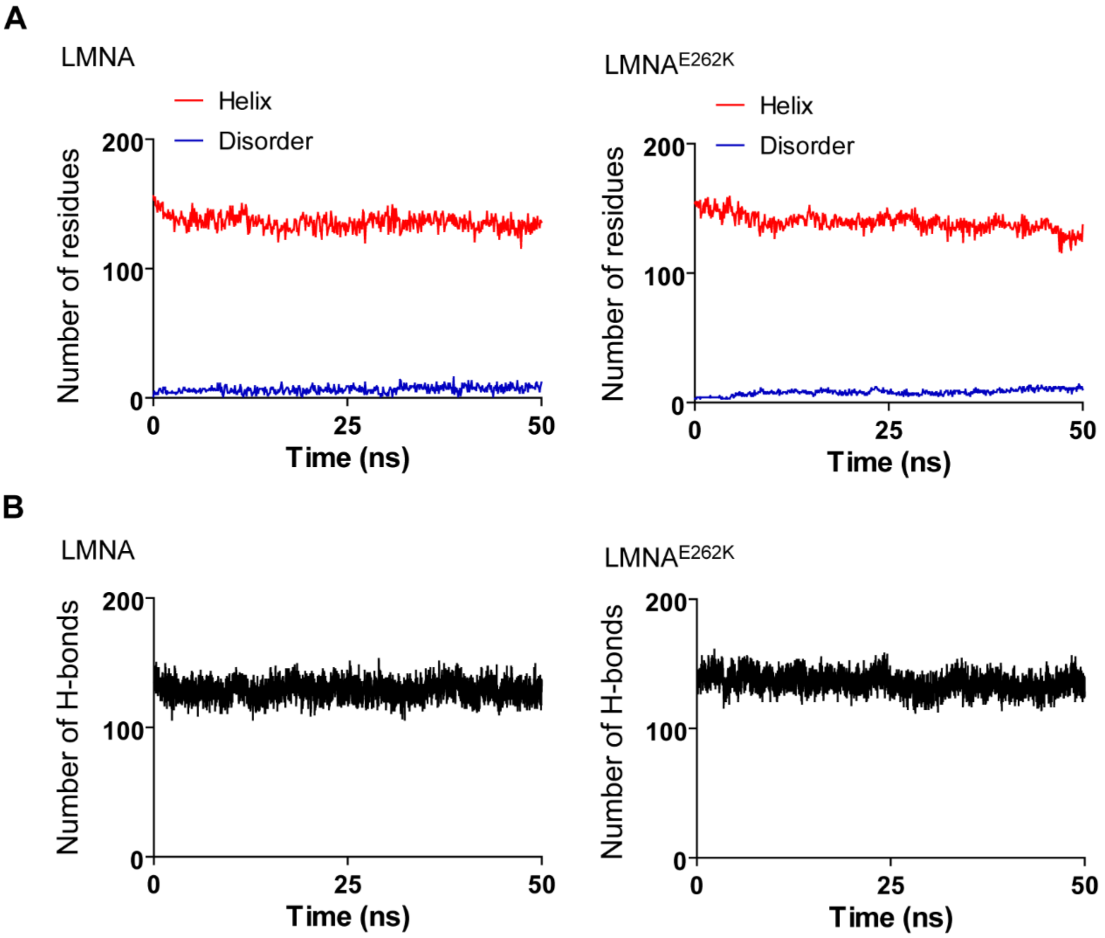

Figure S4. (A) Secondary structure parameter, and (B) hydrogen bonds of LMNA and LMNA^{E262K} over the 50ns molecular dynamics simulation.

Table S3: Variant prioritization and filtering strategy used to analyze exome sequencing data.

Total called variants	2071
Rare variants (<1%)	120
Exonic and splicing variants	120
Homozygous variants	10
Heterozygous variants	110
Variant in concordance with the observed phenotype	1

- Variant frequency equal to or less than 1% in population databases (gnomAD, 1000 Genomes Project and our in-house data of 1659 exomes)
